# Relationships between abiotic factors, foliage chemistry and herbivory in a tropical montane ecosystem

**DOI:** 10.1007/s00442-024-05630-y

**Published:** 2024-10-25

**Authors:** Alejandro de la Fuente, Kara N. Youngentob, Karen J. Marsh, Andrew K. Krockenberger, Stephen E. Williams, Lucas A. Cernusak

**Affiliations:** 1 College of Science and Engineering, James Cook University, Townsville, QLD Australia; 2 Research School of Biology, Australian National University, Canberra, ACT Australia; 3 The Fenner School of Environment and Society, Australian National University, Canberra, ACT Australia; 4 College of Science and Engineering, James Cook University, Cairns, QLD Australia

**Keywords:** Herbivore–plant interaction, Biogeochemical dynamics, Tropical montane ecosystem, Bottom-up regulation, Resource availability

## Abstract

Herbivore–plant interactions are fundamental processes shaping ecosystems, yet their study is challenged by their complex connections within broader ecosystem processes, requiring a nuanced understanding of ecosystem dynamics. This study investigated the relationship between nutrient availability and insect herbivory in the Australian Wet Tropics. Our objectives were threefold. Firstly, to understand what factors influence nutrient availability for plants and herbivores across the landscape; secondly, to investigate how trees of different species respond to nutrient availability; and thirdly, to unravel how the relationships between resources and plant chemistry affect herbivory. We established a network of 25 study sites covering important abiotic gradients, including temperature, precipitation, and geology. Employing a hierarchical modelling approach, we assessed the influence of climate and geology on resource availability for plants, primarily in the form of soil nutrients. Then, we explored the influence of the above factors on the interaction between herbivory and foliage chemistry across three widespread rainforest tree species, comparing how these relationships emerged across genera. Our findings suggest an overarching influence of climate and geology over soil chemistry, foliar nitrogen, and insect herbivory, both directly and indirectly. However, individual constituents of soil fertility showed equivocal influences on spatial patterns of foliage chemistry once site geological origin was accounted for, suggesting a questionable relationship between individual soil nutrients and foliar composition. We have demonstrated that herbivore–plant interactions are complex dynamics regulated by an intricate web of relationships spanning different biogeochemical processes. While our results provide some support to the notion that herbivory is affected by resource availability, different species growing under the same conditions can show differing responses to the same resources, highlighting the importance of identifying specific limiting factors rather than simpler proxies of resource availability.

**Supplementary Information:**

The online version contains supplementary material available at 10.1007/s00442-024-05630-y.

## Introduction

Herbivory is a fundamental ecological process with far-reaching effects on ecosystem functioning. The intricate relationship between herbivores and plants cascades through key ecosystem dynamics, including food webs, community diversity, and nutrient cycling (Coley and Barone 1996; Forrister et al. 2019). Consequently, unravelling the dynamics of herbivory pressure, and the drivers that shape this relationship across the landscape, is crucial to decipher the ecological and evolutionary ramifications of herbivore-plant interactions (Janzen 1974; Whittaker 1979).

In attempting to explain these ecological relationships, researchers often seek clines in herbivore–plant interactions that can be captured across environmental gradients, usually associated with changes in resource availability (Endara and Coley 2011; Moreira et al. 2018; Rasmann et al. 2014). The prevailing hypothesis posits that plants growing in resource-rich environments should be able to tolerate herbivory better than plants growing in more challenging conditions (Maschinski and Whitham 1989; Whitham 1991). This hypothesis stems from the widely accepted notion that herbivory generally undermines plant fitness (Belsky 1986). The intuitive appeal of this hypothesis has laid a robust foundation for developing theories aiming to explain evolutionary links between herbivory pressure and plant defences (Coley and Barone 1996; Coley et al. 1985; Endara and Coley 2011).

While conventional wisdom often suggests a negative correlation between herbivory and environmental stress, the accurate representation of these complex ecological relationships along simplified environmental gradients remains a subject of ongoing debate. For instance, Moles et al. (2011) challenged the prevailing idea of latitudinal gradients shaping herbivory pressure (Coley and Barone 1996), revealing that simple abiotic gradients might fail to predict variable ecological patterns across large scales. Similarly, Wise and Abrahamson (2005) demonstrated the pitfalls of using simplified resource gradients as predictors of herbivory. Their work emphasised the significance of a refined model that explores the multidimensional aspect of resource gradients for a more mechanistic understanding of the links between plant fitness and herbivory pressure (Wise and Abrahamson 2007).

In addition to resource availability, other processes can also influence plant susceptibility to herbivory. These processes can act as regulators, influencing factors that directly affect herbivory pressure, albeit not always exerting a direct control over herbivores themselves (Lynn and Fridley 2019). For example, temperature could play an important role in herbivore–plant relationships by positively impacting insect herbivore metabolism, population abundance and diversity (Moreira et al. 2018; Newell et al. 2023). However, temperature could also affect plant susceptibility to herbivory via influences on plant defences through the regulation of edaphic chemical processes (Lynn and Fridley 2019). Overall, the comprehensive understanding of herbivore–plant interactions in natural environments might be hindered by the complex interplay of co-varying environmental factors across resource availability gradients, potentially leading to confounding effects.

Montane ecosystems are valuable natural environments for studying herbivore–plant interactions. These systems solve part of the challenge of studying variable ecological patterns across environmental gradients, as they offer strong abiotic gradients across a relatively small spatial scale, reducing the susceptibility to potential confounding effects from broad-scale factors (Galmán et al. 2018; Moreira et al. 2018; Pellissier et al. 2012). For instance, environmental variation across elevational gradients occurs independently of variation in day length or inter-annual variation in climate, providing a useful setting to investigate the variability in ecological relationships across space (Galmán et al. 2018).

In this study, we aimed to examine the interplay of environmental gradients in regulating insect herbivory pressure in the Australian Wet Tropics across three widespread rainforest canopy tree species. Specifically, we set out to (1) describe the abiotic drivers shaping resource availability for plants expressed through climate and soil nutrients; (2) understand plant responses to abiotic gradients in terms of variability in foliage chemistry; (3) investigate potential correlations between insect herbivory damage, foliage nutritional chemistry and abiotic factors; (4) compare tree species to discern contrasting responses to abiotic gradients owing to differences in potential limiting resources; and (5) unravel the indirect influence of abiotic factors on the different processes under investigation.

We predict an overarching effect of geology and climate on soil nutrients across the landscape given the strong elevational and geological gradient that defines montane ecosystems (Weil and Brady 2017; Singh Ramesh et al. 2023; Galmán et al. 2018; Moreira et al. 2018). In addition to the direct effect of abiotic factors over resource gradients, we anticipate climate and geology to propagate their influence across the ecosystem, indirectly impacting herbivore–plant interaction through their potential influence on soil nutrient availability and foliage nutritional chemistry (Lynn and Fridley 2019). If there is a direct relationship between soil and plant chemistry, then variability in plant chemistry should reflect the distribution of soil nutrients across the landscape. However, pioneer and late successional species will likely respond differently to these resource gradients, potentially reflecting their sensitivity to different limiting factors (Wise and Abrahamson 2007, 2005). Furthermore, we predict herbivory pressure to intensify with increasing resource availability due to the expected greater potential compensation for herbivory and lower investment in defences in resource-rich environments (Whitham 1991; Coley et al. 1985; Endara and Coley 2011).

To address these aims, we first identified the main factors that could influence the variability in insect herbivory pressure in our study system: climate, geology, soil nutrients, and foliage nutritional chemistry. Then, we explicitly connected these components in a hierarchical model to study three main processes: (1) the climatic and geological influence on soil nutrients, (2) plant–soil relationships, and (3) interaction between amount of herbivory and plant nutritional chemistry. Following this integrated approach, we assessed the influence of the different resource gradients and climate on herbivory, both directly and indirectly.

## Materials and methods

### Study system

**Fig. 1:**
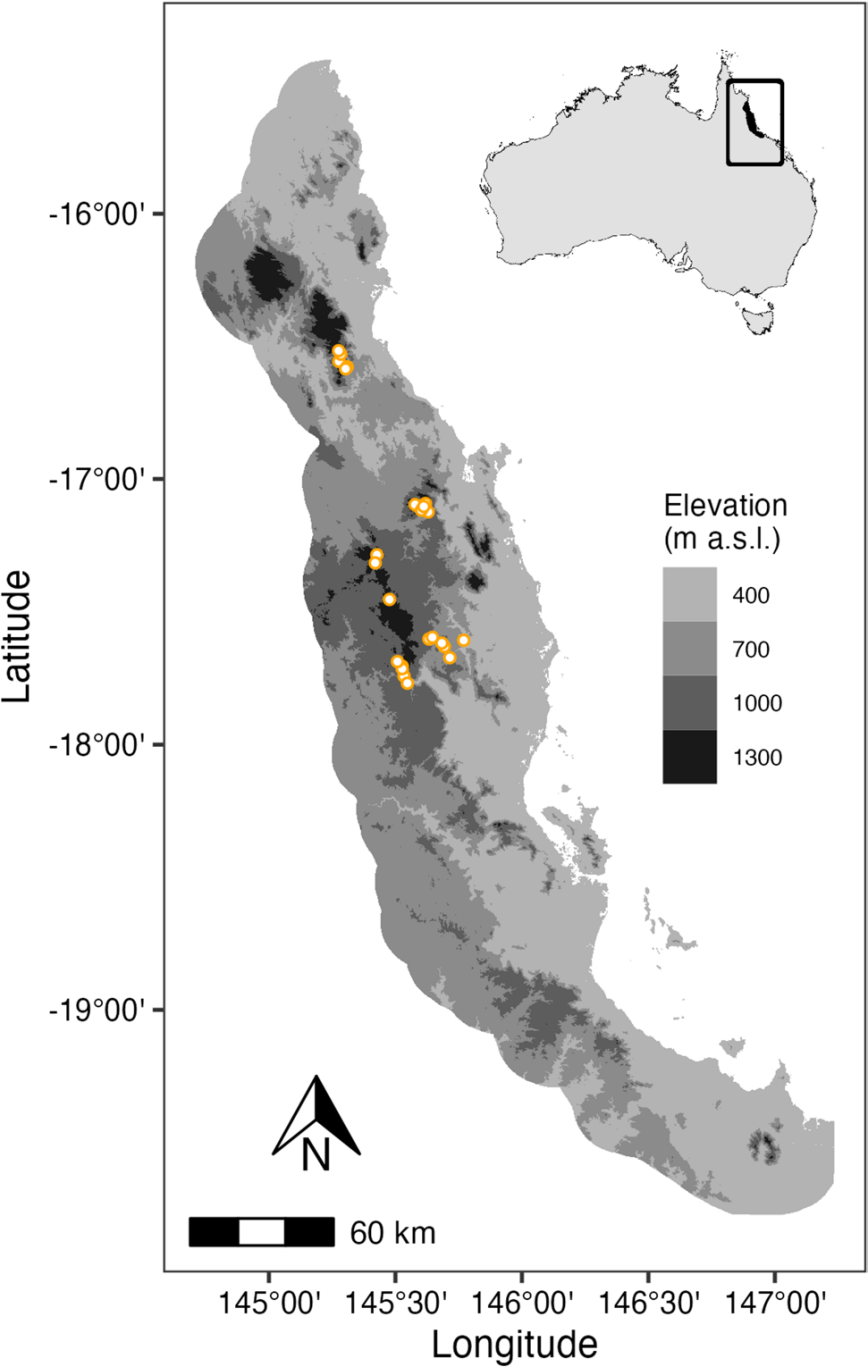
The Australian Wet Tropics bioregion and its location in Australia. The grey colour palette represents the elevational gradient of the region. Points depict the location of the 25 study sites

The Australian Wet Tropics ecosystem, situated along the northeast coast of Queensland (Fig. 1), comprises an area of approximately 7000 km^2^. The bioregion is characterised by a rugged landscape covered primarily by mixed tropical rainforests. Notophyll rainforest is the most extensive vegetation type, followed by mesophyll rainforest, predominantly below 400 m in elevation, and microphyll rainforest on the upper slopes and higher peaks.

The mountain ranges that shape the bioregion create a significant elevational gradient crucial in shaping biodiversity patterns and ecological processes (Williams et al. 1995; Singh Ramesh et al. 2023). Elevation ranges from sea level to highlands at 1000 m, with isolated peaks reaching approximately 1620 m. Consequently, the mountain ranges in the Australian Wet Tropics define a pronounced thermal gradient spanning between 14 and 26 °C in mean annual temperature. The topographic features of the region also impose a longitudinal precipitation gradient, with higher rainfall near the coast (around 4000 mm annually) and decreasing towards the western extent of the tropical rainforest distribution (approximately 1200 mm annually). This abrupt change in rainfall occurs within 70 km of the coastline, emphasising the great variability in abiotic conditions on a relatively small spatial scale. Additionally, soil chemistry in the Wet Tropics varies markedly due to the diverse geological origins of the region. The soils primarily derive from granites, igneous rocks such as rhyolites and basalts, and metamorphic sedimentary rocks. As a result, the bioregion presents a pronounced edaphic gradient, ranging from deep and nutrient-rich basalt-derived soils to shallow and nutrient-poor lithosols (Isbell 2016).

To capture the range of climatic and edaphic gradients of the Australian Wet Tropics, a network of 25 study sites, each approximately 1 ha, was established in notophyll rainforests, the predominant vegetation formation in the region. These study sites cover 94% of the climatic variability in the Australian Wet Tropics (Williams et al. 2010) and span an elevation range between 400 and 1300 m. Moreover, the study site network covered the predominant soil parent material found across the mountain ranges of the Australian Wet Tropics, namely basalt, rhyolite, and granite. This study design allows for a robust examination of ecological interactions across the diverse environmental conditions present in the region. Further information about site location, geology, and climate is provided in Supplementary materials.

### Study species

To investigate patterns in insect herbivore-plant interactions across the abiotic gradients defined above, we selected a set of canopy tree species based on their high abundance and wide distribution (see Table 1). These selection criteria were not intended to represent the region’s rich tree biodiversity but to encompass the desirable biogeographical characteristics that allowed us to investigate the variability of ecological interactions across the landscape. Our species selection included both fast-growing generalist and slow-growing montane rainforest tree species from different families to account for potential differences in resource allocation associated with different growth strategies (Coley et al. 1985; Fine et al. 2004). However, late successional species tend to have a more constrained geographical range than the more generalist pioneer species, resulting in an uneven sampling coverage across the abiotic gradients under investigation (Table 1). To cope with the differing distributional patterns when comparing the emergent relationships between growth strategies, we included two closely related species from the genus *Flindersia* to expand the sampling coverage along the abiotic gradients for late successional species (Table 1). Despite its limitations, this approach enabled inferring the effect of biogeochemical processes on foliage nutritional chemistry and herbivory within and between species with different growth strategies.

**Table 1:** Overview of the elevational range, habitat preference, growth strategy, sample size, and site coverage for the selected canopy tree species

Species name	Family	Elevational range (m a.s.l.)	Habitat	Sample size	Site coverage
*Alphitonia petriei*	Rhamnaceae	0–1300	Generalist; Rainforest regrowth; Pioneer species	115	100%
*Flindersia brayleyana*	Rutaceae	0–1150	Well-developed montane rainforest; Late successional species	22	48%
*Flindersia pimenteliana*	Rutaceae	0–1300	Well-developed montane rainforest; Late successional species	42	60%
*Flindersia* species combined	Rutaceae	0–1300	Well-developed montane rainforest; Late successional species	64	84%

### Climate data

The climatic data (temperature and precipitation) was obtained from the “accuCLIM” climate layers developed for the Australian Wet Tropics (Storlie et al. 2013). These climate layers downscale gridded spatial climate data at 250 m resolution from empirical weather data collected between 2006 and 2009 at 54 rainforest sites, offering a fine-grained spatial representation of the bioregion’s climate. Our model incorporated climate variables as linear and quadratic terms, accommodating potential non-linear relationships that may emerge across the elevational gradient (Moreira et al. 2018).

Given the strong climatic gradient imposed by the orography of the bioregion, we expected climate to play a central role in the regulation of biogeochemical cycles (Weil and Brady 2017; Austin and Sala 2002; Ren et al. 2015; Wright et al. 2003; Wright and Westoby 2002). Additionally, we anticipated that climate would directly affect herbivory pressure through its influence on insect herbivore populations and diversity (Galmán et al. 2018; Newell et al. 2023).

### Soil chemistry data

The characterisation of soil chemistry was carried out at the site level. Our sampling design aimed to capture the heterogeneity in soil composition, deriving the average conditions to which trees were exposed at each location. We randomly selected six, 150 m transects at each site with a minimum separation of 200 m between them. Along these transects, we collected soil cores every 50 m using a 65 mm Jarret-style auger. To account for the vertical variation in soil chemistry, we collected soil cores at three different depths at each sampling point (0–10 cm, 20–30 cm, and 50–60 cm). For each site and depth, samples were homogenised in the field, resulting in a total of three samples per study location. Samples were sent to the Chemistry Centre (Department of Environment and Science, Queensland Government, Brisbane, Australia) for chemical analysis promptly after collection (< 5 days).

Soil components analysed included total nitrogen and phosphorus (Kjeldahl digestion), exchangeable cations (Ca, Mg, Na, K), and organic carbon (Dumas combustion). To establish a comparable measure of soil elements across sites, we computed the mean value of each element across the vertical gradient, partly accounting for the potential effect of soil depth on nutrient acquisition (Göransson et al. 2006). In alignment with the study’s overarching goal of understanding the interconnections within the system, our investigation focussed on specific soil elements of significant relevance in biogeochemical cycles that could, directly and indirectly, affect herbivory pressure. These include nitrogen and phosphorus (Peng et al. 2021; Read et al. 2008; Westerband et al. 2023; Wright et al. 2005), potassium (Santiago et al. 2012), sodium (Lynn et al. 2023; Kaspari 2020), and the ratio between carbon and nitrogen (Singh Ramesh et al. 2023; Weil and Brady 2017).

### Foliage nutritional chemistry data

Measures of foliage nutritional quality for herbivores often consider the influence of nitrogen (N) as an important limiting factor due to its relatively low concentration in plant tissues (White 2012). However, the total nitrogen content in leaves may not accurately reflect the availability of this limiting factor for all herbivores. Although this study focused on insect herbivory, we also included a measure of foliar nitrogen that incorporates the influence of tannins on nitrogen digestibility (Wallis et al. 2012). Insects are unlikely to be affected by tannin binding due to the dissociation of tannins from proteins in the alkaline environment of insect guts (Salminen and Karonen 2011); however, the inclusion of nitrogen digestibility remains valuable in this study as it could reflect levels of tree investment in defences in the form of protein-binding tannins along abiotic and resource availability gradients.

Prior to chemical analyses, foliage samples were freeze-dried and ground to pass through a 1 mm particle-size sieve. Ground samples were scanned on a Foss XDS near-infrared reflectance spectrometer. Chemical assays were performed on a representative subset of samples, comprising 33% of our sample size, which was selected based on its near-infrared absorption spectrum and identified using Mahalanobis distance. Calibration equations were developed to predict values of foliar chemistry based on the spectra and reference values obtained from the chemical assays, following the approach outlined by Ebbers et al. (2002). This method allowed for a rapid, cost-effective, and non-destructive assessment of foliage nutritional chemistry by establishing a relationship between spectral information and nitrogen content (Youngentob et al. 2012). The calibration equations derived from this process led to a robust prediction of total nitrogen (*R*^2^ = 0.98, standard error of cross-validation [SECV] = 0.06), nitrogen digestibility (*R*^2^ = 0.96, SECV = 0.1259), and dry matter digestibility (*R*^2^ = 0.91, SECV = 3.68). Further details about the spectral transformations and the development of the calibration equations can be found in Youngentob et al. (2012).

To measure nitrogen digestibility, we followed the method outlined by DeGabriel et al. (2008). Briefly, this method consists of an in vitro digestion where paired samples are incubated in a series of buffers and enzymes. Total nitrogen and non-digestible nitrogen were measured in a Truspec Carbon/Nitrogen Analyser (LECO Corp, St. Joseph, MI, USA), using the pre- and post-digestion samples, respectively. The index of nitrogen digestibility used in this study was then calculated as a function of the change in nitrogen and dry matter, determined by comparing samples’ weight and nitrogen concentration before and after digestion, with a high index indicating a high percentage of available nitrogen relative to the total nitrogen content in the foliar tissue. Further details on the calculation of available nitrogen and lab protocol have been extensively covered in DeGabriel et al. (2008) and Wallis et al. (2012).

$$\text{N } \text{digestibility}= \frac{\left({\text{N}}_{0} * {\text{DM}}_{0}\right)-\left({\text{N}}_{1} * {\text{DM}}_{1}\right)}{\left({\text{N}}_{0} * {\text{DM}}_{0}\right)}$$

$$\text{Dry matter digestibility}= \frac{\left({\text{DM}}_{0}\right)-\left({\text{DM}}_{1}\right)}{\left({\text{DM}}_{0}\right)}$$

where DM stands for dry matter and the subscript indices represent pre-digestion (*X*_0_) and post-digestion (*X*_1_).

### Insect herbivory data

Insect herbivory pressure was quantified as the proportional damage or tissue loss from attached leaves. Leaves were collected from 120 canopy trees (sample height: 24.71 ± 6.28 m) during the dry seasons (April–November) of 2021 and 2022, following the methods outlined by Youngentob et al. (2016). The study’s scale required tree sampling to span two seasons, as sampling all sites in a single season was not feasible. While foliar chemical properties can vary over time, the traits measured here have been reported to be relatively stable across similar seasons (Steinbauer et al. 2015). Thus, to minimise potential variability in these traits, we sampled during the same season in both years. Additionally, because each tree was sampled only once, temporal variability across individuals was not accounted for in the model.

Based on preliminary analyses using a reference sample (data not shown), we determined that the sampling of 20 leaves per individual tree provided an adequate sample size to measure herbivory, ensuring a complete range of apparent foliage damage across species (i.e., damage values reached a stable normal distribution within samples). To achieve the desired sample size for each tree, we randomly collected approximately 40 mature leaves from retrieved canopy branches. Half the leaves were haphazardly selected for herbivory damage measurement using the “leafbyte” image processing software (Getman-Pickering et al. 2020). Herbivory damage was measured only on mature leaves in this study due to the low availability of young leaves for slow-growing montane species at the sampling time. The remaining half of the leaves were snap-frozen in the field using dry ice at − 80 °C to preserve their chemical composition for subsequent analyses.

We focussed on quantifying the proportional leaf loss to herbivory without distinguishing the specific types of damage (i.e., chewing, sucking, or mining). This approach provided us with a general measure of insect herbivory pressure at the tree level, allowing for an assessment of the overall impact of insect herbivores on the study tree species.

### Statistical analysis

**Fig. 2:**
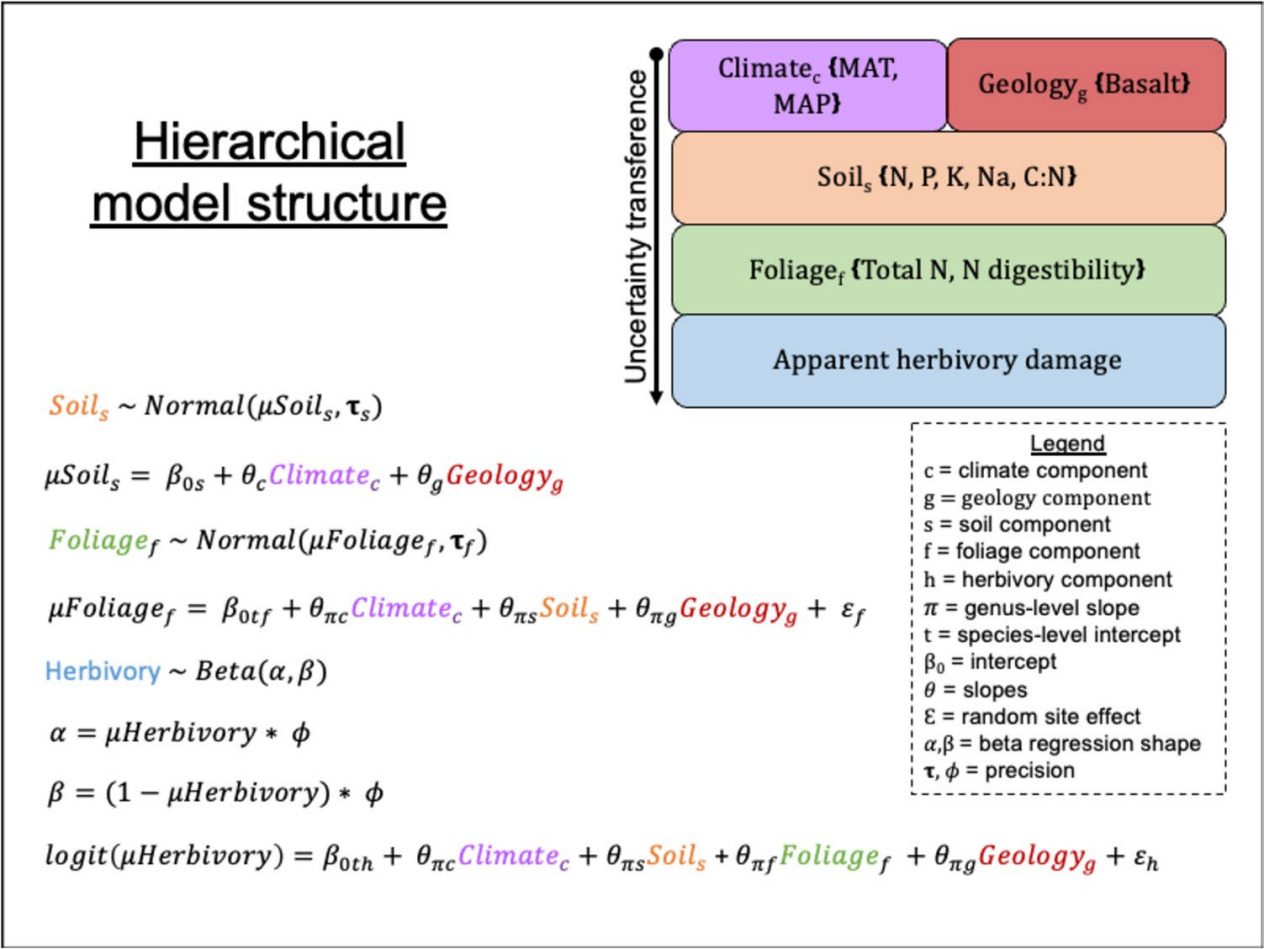
Overview of the hierarchical model developed to study herbivory pressure across environmental gradients. Colours represent the main gradients in the system. *P* phosphorus, *N* nitrogen, *C:N* carbon–nitrogen ratio, *K* potassium, *Na* sodium, *MAT* mean annual temperature, *MAP* mean annual precipitation

We employed a statistical model that aimed to reflect the interconnectivity of the system by integrating climate, geology, soil element concentrations, foliage nutritional chemistry, and herbivory damage data. Our approach involved the development of a hierarchical model that mirrored the natural structure of the study system (Fig. 2). This model enabled us to disentangle the effects of each environmental gradient on important emergent processes, such as (1) the influence of climate and geology on soil chemistry, (2) plant–soil relationships, and (3) herbivore–plant interactions. Furthermore, the hierarchical structure of the model allowed us to examine the influence of climatic and edaphic gradients throughout the ecosystem, revealing the potential impact of these abiotic factors on herbivory pressure, both directly and through the control of intermediate processes. We accounted for the inherent variation in foliar chemistry and herbivory damage across plant species by implementing species-specific intercepts (Volf et al. 2022; Forrister et al. 2023). Additionally, by segregating slopes based on genus, we were able to compare the effect of different abiotic gradients on the processes under investigation (see “Study species”). This combination resulted in foliage and herbivory models with a structure defined by three intercepts and two slopes per predictor (Fig. 2). A summary of the data structure and sample size for each sub-model is provided in Supplementary materials.

We implemented our model in a Bayesian framework. Markov Chain Monte Carlo methods were used to sample the joint posterior distribution (Thompson and Brooks 2003) using JAGS (Plummer 2003), implemented with the jagsUI R package (Kellner 2021; R Core Team 2021). We reflected the absence of prior information about model parameters by implementing vague priors (see JAGS code in Supplementary materials for details). Additionally, we accounted for extra unexplained heterogeneity across sites by including a random site effect. Foliage and soil components were normally distributed, while herbivory pressure (percentage of leaf eaten) followed a beta distribution. We ran three parallel chains of 400,000 interactions, used half as burn-in, and thinned the rest by one in 100 to obtain 6000 samples of the joint posterior distribution. The sampling process led to robust parameters convergence ($\widehat{R}-1<0.1$, (Gelman and Hill 2006)). We further inspected trace plots to validate good mixing and investigated the posterior predictive check by plotting the residual sums of squares for observed and simulated data. Good fitting models are expected to have values falling on a 1:1 line, suggesting a similar error structure between the observed data and the data simulated by the model (Figure S2). We paired the posterior predictive checks with the estimation of Bayesian *p*-values, with values close to 0.5 (Figure S2) indicating adequate model fit (Gelman et al. 1996). Model structure and variable selection are explained in more detail in the Supplementary materials.

## Results

### Spatial variability in soil nutrients

**Fig. 3:**
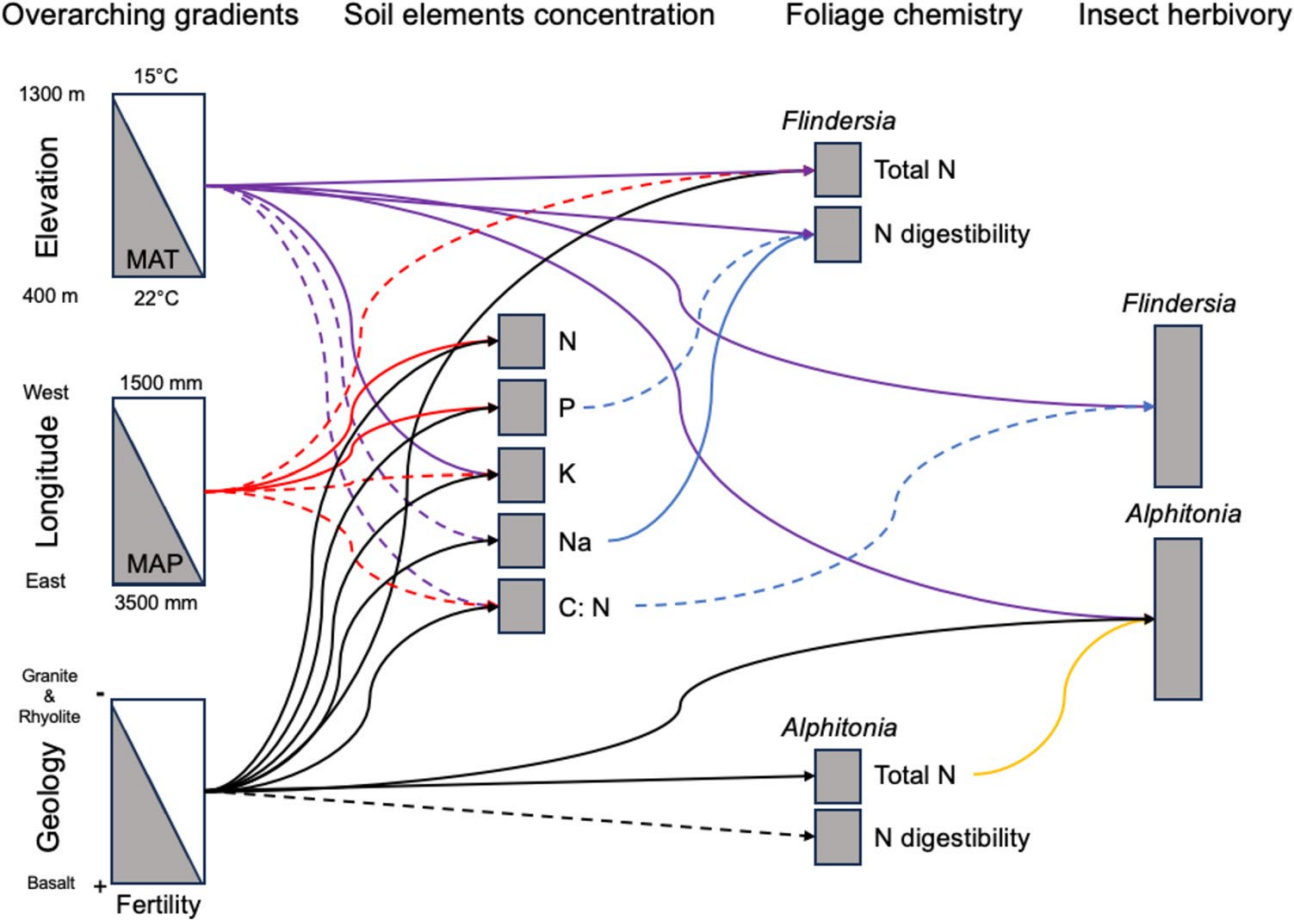
Pathways of emergent connections in the Australian Wet Tropics. This plot represents the direct and indirect associations in the ecosystem. For simplicity, only relationships that do not greatly overlap zero are included. Dashed lines represent negative relationships, while solid lines show positive associations. Colours segregate the effect of each major component in the model to ease interpretation. *P* phosphorus, *N* nitrogen, *C:N* carbon–nitrogen ratio, *K* potassium, *Na* sodium, *MAT* mean annual temperature, *MAP* mean annual precipitation. Relationships showing the overlap between the predictors and raw data are provided in figure S3–10

**Fig. 4:**
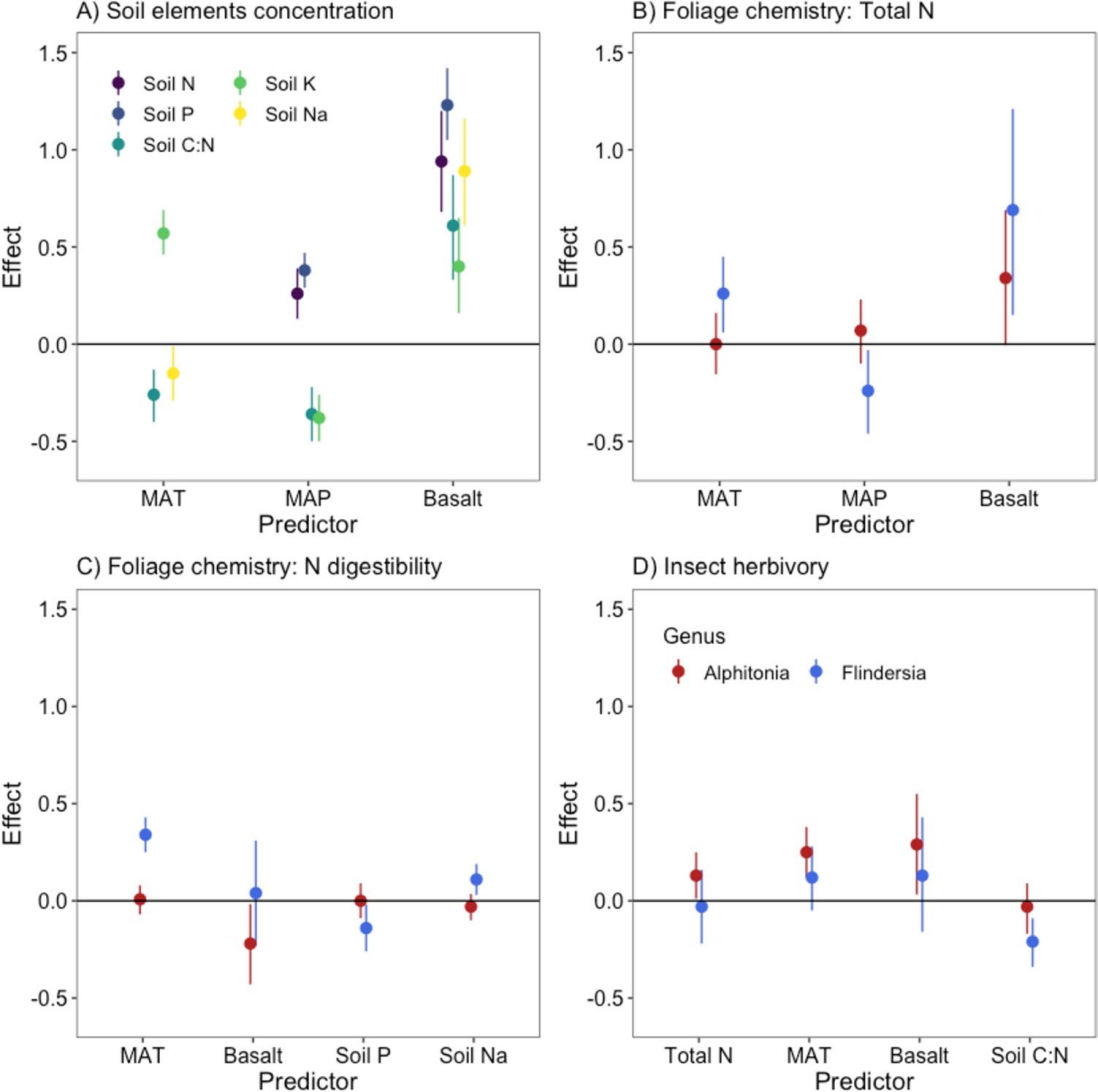
Summary plot showing the effects of different drivers on soil element concentration (**A**), total foliar N (**B**), N digestibility (**C**), and insect herbivory pressure (**D**). Values represent the posterior mean and 89% credible interval (McElreath 2020). Colours represent different soil elements in panel A and different tree genera in panel **B**–**D**. *P* phosphorus, *N* nitrogen, *C:N* carbon–nitrogen ratio, *K* potassium, *Na* sodium, *MAT* mean annual temperature, *MAP* mean annual precipitation. Relationships showing the overlap between the predictors and raw data are provided in figure S3–10

Soil nutrient availability was explained by the interplay among elevation, longitude, and geology, the predominant environmental gradients shaping the bioregion (Fig. 3; see “Study site”). Geology, defined by the parent materials, influenced soil element concentrations, suggesting higher nutrient concentration in sites of basaltic origin than in those derived from granite or rhyolite rocks (Figs. 3, 4A). Soil N and P increased with higher precipitation, while soil K and C:N decreased along the same gradient (Figs. 3, 4A). Temperature showed a positive relationship with soil K but a negative association with soil Na and soil C:N (Figs. 3, 4A).

### Foliage nutritional chemistry across resource gradients

We found diverging patterns in the variability of foliar chemistry across species. Specifically, *Alphitonia* showed lower N digestibility than *Flindersia*, with *Flindersia pimentaliana* exhibiting the greatest level of N digestibility across the species examined (Table S2). Conversely, total N was present in higher concentration in *Alphitonia* than in *Flindersia*, with *Flindersia brayleyana* showing the lowest level of total N across the study species (Table S2).

The concentration of total N across trees was significantly influenced by geology, with individuals growing in basaltic soils showing greater levels of total N than trees growing in granite and rhyolite soils. This pattern remained consistent across *Alphitonia* and *Flindersia* species (Figs. 3, 4B). N digestibility was negatively correlated with soil P for *Flindersia* and basaltic soils for *Alphitonia* (Figs. 3, 4C). A diverging pattern of N digestibility between *Alphitonia* and *Flindersia* was related to their response to soil Na, with *Flindersia* suggesting a positive association, while *Alphitonia* showed a marginal response (Fig. 4C). *Flindersia* foliar chemistry also exhibited significant sensitivity to climate, suggesting an increase in total N and N digestibility with increasing temperatures and a decrease in total N with higher rainfall (Figs. 3, 4B, C).

### Patterns in herbivory damage

Herbivory pressure, measured as the proportional tissue loss from attached leaves, was low across the trees sampled, with average damage close to 4% (although individual leaf values ranged from 0.002 to 54.79%). The variability in herbivory showed no clear distinction between *Alphitonia petriei* (average = 2.5–3.3%) and *Flindersia pimentaliena* (average = 1.9–2.7%; Table S2). In contrast, *Flindersia brayleyana* showed generally higher levels of herbivory (average = 4.3–9.4%; Table S2) than *Alphitonia petriei* and *Flindersia pimentaliena*.

The variability in herbivory pressure across the landscape was influenced to some degree by all abiotic gradients but not consistently across species (Fig. 3). *Alphitonia* showed higher levels of herbivory in sites on basalt, higher temperature, and across trees with higher total foliar N (Fig. 3). In a similar way, there was a negative relationship between herbivory and soil C:N and a positive relationship between herbivory and temperature for *Flindersia* species (Figs. 3, 4D). However, we note that the effect of temperature on herbivory for *Flindersia* showed greater uncertainty (Fig. 4D; Figure S3).

### Indirect effects

In addition to the direct effects described in the sections above, our result showed a variety of potential intermediate processes that emerged from the hierarchical structure of the system. Precipitation indirectly influenced N digestibility and herbivory pressure for *Flindersia* through its influence on soil P and soil C:N, respectively (Fig. 3). Temperature, in addition to the direct relationship to foliar chemistry and herbivory pressure (Fig. 4), also impacted indirectly N digestibility and herbivory damage for *Flindersia* by influencing soil Na and soil C:N, respectively. Lastly, geology was positively associated to herbivory damage for *Alphitonia* both directly and through its correlation to total foliar N (Fig. 3).

## Discussion

In this study, we have delved into the relationship between herbivory and resource availability across three widespread rainforest tree species in a tropical montane ecosystem, the Australian Wet Tropics. Our study has placed a particular emphasis on investigating herbivore-plant interactions within the broader context of biogeochemical dynamics. This approach has allowed us to investigate (1) what factors affect resource availability across multiple abiotic gradients, (2) how plant chemistry responds to the variation in resources, and (3) how the interplay between resource gradients and foliage nutritional chemistry correlates to patterns in insect herbivory across the landscape. Overall, our findings contribute to a deeper understanding of the intricate relationships between environmental factors, plant chemistry, and herbivory dynamics in tropical montane ecosystems.

### Patterns in resource availability across abiotic gradients

The climatic and geological gradients that occur across the Australian Wet Tropics showed a strong relationship to all the factors we examined. Specifically, our results suggested an interplay between climate and geology in shaping biogeochemical dynamics in montane ecosystems (Singh Ramesh et al. 2023; Weil and Brady 2017). Along the elevational gradient, some measured soil chemical constituents were significantly influenced by temperature. We found evidence of a negative plant-soil relationship at higher elevations, possibly related to the temperature-induced slowdown in soil microbial activity and nitrogen mineralisation (Weil and Brady 2017; Salinas et al. 2011). Across the longitudinal gradient, soil nutrient availability was primarily influenced by changes in rainfall. Notably, key soil nutrients, such as N and P, increased with higher precipitation, emphasising the role of rainfall in influencing nutrient availability (Austin and Sala 2002; Ren et al. 2015). However, rainfall could also negatively influence the concentration of some soil elements through leaching, suggested in this study by the relationship between precipitation and soil K. Independently of climatic conditions, geology showed a strong relationship to soil nutrient levels, stressing the important role of parent materials in determining the supply of rock-derived soil elements (Anderson 1988), although it was notable that soil chemical constituents did not consistently influence foliar chemistry, as is often assumed.

### Effects of resource availability on foliage nutritional chemistry

Foliar nutritional chemistry did not consistently correspond with broader biogeochemical processes. Despite our findings suggesting that geology was strongly correlated to soil nutrients, the positive relationship between basaltic soils and foliar nutritional chemistry could not be explained by individual constituents of soil fertility. This discrepancy might reflect the importance of understanding soil fertility as a multidimensional factor shaped by the interaction between soil nutrients and other physical or chemical properties that are associated with the underlying site geology. While understanding soil fertility as a multidimensional factor offers an intuitive explanation for the observed patterns (Millard 1993; Norris et al. 2013), it is important to acknowledge the potential influence of external variables that co-vary with geology, potentially impacting spatial variation in foliage nutritional chemistry.

In addition to the influence of geology on foliage nutritional chemistry, we found a significant climatic influence (MAP and MAT) on foliar N for *Flindersia* but not for *Alphitonia*, which could reflect differences in geographical restrictions between these genera. While the generalist *Alphitonia* is widespread and well adapted to a diverse range of climatic conditions (Hyland et al. 2010), the more geographically constrained *Flindersia* could suffer greater fitness limitation along climatic gradients, thereby showing reduced foliar N in less favourable environments (Singh Ramesh et al. 2023; Singh Ramesh 2023). Moreover, our results suggested an intriguing pattern related to the relationship between N digestibility and resource gradients, where N digestibility decreased with increased soil P for *Flindersia* and basaltic soils for *Alphitonia* (Fig. 3). This pattern did not align with our initial expectations. We propose two potential explanations for this negative relationship between N digestibility and nutrient availability.

The negative relationship between N digestibility and nutrient availability observed in this study could be related to resource-driven plant investment in defences. In resource-rich environments, plants might allocate more resources towards defences against herbivory, such as carbon-based compounds like tannins, compared to plants growing in more resource-limited conditions (Hahn and Maron 2016; Moreira et al. 2018; López-Goldar et al. 2020). This process would result in a decrease in N digestibility but not in total N in high-resource environments, aligning with the patterns we observed in this study. An alternative explanation could be related to the presence of alkaloids in the tree species included in this study (Robertson et al. 2018; Al Omar et al. 2022), which could suggest that our measure of foliar N would capture not only protein content but also alkaloid concentration. These potential relationships warrant further investigation.

### The drivers of herbivory damage

The low value in herbivory damage found in this study markedly contrasts with findings in other tropical wet forests, for which insect herbivory damage has been reported to reach values of up to 48% (Coley and Barone 1996). These differences could be attributed to a number of factors, including (1) the discrete nature of the measurement taken in this study, as opposed to continuous monitoring, preventing the determination of total leaf loss (Lowman 1985); (2) the consideration of only mature leaves, which may be less consumed by insect herbivores than young leaves (Coley and Barone 1996); (3) the lower herbivory damage on the canopy stratum of the forest, highlighting the shade/sun differences in herbivory pressure (Lowman 1985); and (4) the potential depression of insect herbivores during the dry season, when our sampling took place (Wolda 1978; Lowman 1985). While including sampling during the wet season and covering various leaf ages and forest strata could potentially yield a higher herbivory rate, our consistent sampling design provides robust insights into spatial variations in herbivory damage across multiple abiotic gradients. Additionally, it offers a comparative framework to assess how these gradients affect species differently, as it is not confounded by changes in seasonal herbivore activity along the forest stratum or differing seasonal patterns in leaf flushing across species.

Our results suggest that herbivory damage was significantly influenced by the same abiotic factors that affected nutrient availability for soils and plants, providing some support for the compensatory continuum hypothesis, where the degree of herbivore impact changes as a function of resource availability (Maschinski and Whitham 1989; Whitham 1991). However, we found differing relationships between herbivory and other measured variables among tree species, suggesting potential contrasts in factors that affect herbivory (Wise and Abrahamson 2005, 2007). For the pioneer species *Alphitonia petriei*, herbivory damage was primarily influenced by geology and its relationship to total foliar N (Fig. 3), with trees growing in soils originated from basaltic rocks showing greater herbivory damage and higher foliar N than trees growing in lithosols. For late successional species of the genus *Flindersia*, soil nutrient levels impacted herbivory pressure via the soil C:N ratio, indicating a decrease in herbivory in sites with slow N mineralisation (Weil and Brady 2017). Although these findings demonstrate some relationship between soil chemistry and the foliar nutritional landscape (Wardle et al. 2004), it is also notable that this relationship was not consistent across plant species. Moreover, many of the soil nutrients commonly associated with soil fertility, including N and P, showed no direct relationship to total foliar N in any species. Consistently, however, herbivore–plant interactions were significantly impacted by climate. Temperature had a strong positive relationship to herbivory for all species included in the study, potentially reflecting the positive effect of temperature on insect physiology, density, and richness along the elevational gradient (Galmán et al. 2018; Moreira et al. 2018).

### Revealing hidden connections

Our analytical approach indicated that the significant effect of abiotic factors on foliage nutritional chemistry and herbivory pressure was partly explained by the cascading effect that climate and geology have on other abiotic and biotic aspects of the system (Lynn and Fridley 2019). A relevant example from our system relates to the indirect relationship between precipitation and herbivory damage in *Flindersia* species, where the effect of rainfall was only detected via its influence on soil C:N (Fig. 3).

## Conclusion

Here, we have demonstrated that herbivore–plant interactions are complex dynamics regulated by an intricate web of relationships spanning different biogeochemical processes. While our results provide some support to the notion that herbivory is affected by resource availability, different species growing under the same conditions can show differing responses to the same resources, highlighting the importance of identifying specific limiting factors rather than simpler proxies of resource availability (Wise and Abrahamson 2005). We also acknowledge the potential for other factors that could contribute to the spatial difference in herbivory, such as the composition of neighbourhood individuals (Kim and Underwood 2015) or plant size/apparency (Chew and Courtney 1991), which were not considered in this study. Furthermore, the regulation of herbivore-plant relationships across the landscape does not rest solely on the direct effect of limiting resources but also on the factors that regulate resource availability. These otherwise hidden links are critical for the understanding of ecosystem dynamics and should be considered when studying ecological relationships, offering a more comprehensive appreciation of the processes that govern the natural world.

## Supplementary Information

Below is the link to the electronic supplementary material.

Supplementary file1 (DOCX 19 KB)

Supplementary file2 (DOCX 6286 KB)

## Data Availability

The data that support the findings of this study are openly available in Dryad at doi: 10.5061/dryad.d51c5b08s.

## Code availability

The JAGS code to recreate the models is included in Supporting information.
