# Supplementary material for: Relationships between abiotic factors, foliage chemistry and herbivory in a tropical montane ecosystem
Source: Oecologia. 2024 Oct 25;206(3-4):293–304. doi: 10.1007/s00442-024-05630-y (PMC11599541; doi:10.1007/s00442-024-05630-y)
Supplement: Supplementary file 1 — Supplementary file1 (DOCX 19 KB) [file 442_2024_5630_MOESM1_ESM.docx]

JAGS model

############

# Likelihood #

############

for(i in 1:n) {

# Herbivory model

y[i] ~ dbeta(alpha[i], beta[i])

alpha[i] <- mu[i] * phi

beta[i] <- (1-mu[i]) * phi

logit(mu[i]) <- a_hr[spp_intercept[i]] + ### intercepts

### Climate

b.mat_hr[genus_slope[i]] * mat[i] +

### Soil

b.cn_hr[genus_slope[i]] * cn[i]+

### Geology

b.basalt_hr[genus_slope[i]] * basalt[i]+

### Foliage

b.totalN_hr[genus_slope[i]] * total_N[i]+

### Random effect

r.site[site[i]]

# residuals (for posterior predictive check)

res.herb[i] <- pow((y[i] - mu[i]),2)

y.new[i] ~ dbeta(alpha[i], beta[i])

res.herb.new[i] <- pow((y.new[i] - mu[i]),2)

# log-lokelihood (for model comparison)

hr_loglik[i] <- logdensity.beta(y[i], alpha[i], beta[i])

# Foliage model

# Sub-model N digestibility

availN[i] ~ dnorm(mu_avail[i], prec_avail)

mu_avail[i] <- a_availN[spp_intercept[i]]+ ### intercepts

### Climate

b.mat_availN[genus_slope[i]] * mat[i] +

### Soil

b.p_kj_availN[genus_slope[i]] * p_kj[i] +

b.na_availN[genus_slope[i]] * na[i]+

### Geology

b.basalt_availN[genus_slope[i]] * basalt[i]+

### Random effect

eps_availN[site[i]]

# residuals (for posterior predictive check)

res.availN[i] <- pow((availN[i] - mu_avail[i]), 2)

availN.new[i] ~ dnorm(mu_avail[i], prec_avail)

res.availN.new[i] <- pow((availN.new[i] - mu_avail[i]),2)

# log-lokelihood (for model comparison)

availN_loglik[i] <- logdensity.norm(availN[i], mu_avail[i], prec_avail)

# Sub-model total_N

total_N[i] ~ dnorm(mu_total_N[i], prec_total_N)

mu_total_N[i] <- a_total_N[spp_intercept[i]]+ ### intercepts

### Climate

b.map_total_N[genus_slope[i]] * map[i] +

b.mat_total_N[genus_slope[i]] * mat[i] +

### Geology

b.basalt_total_N[genus_slope[i]] * basalt[i]+

### Random effect

eps_total_N[site[i]]

# residuals (for posterior predictive check)

res.total_N[i] <- pow((total_N[i] - mu_total_N[i]),2)

total_N.new[i] ~ dnorm(mu_total_N[i], prec_total_N)

res.total_N.new[i] <- pow((total_N.new[i] - mu_total_N[i]),2)

# log-lokelihood (for model comparison)

totalN_loglik[i] <- logdensity.norm(total_N[i], mu_total_N[i], prec_total_N)

# Soil model (likelihood)

# Submodel Soil N

n_kj[i] ~ dnorm(mu_n_kj[i], prec_n_kj)

mu_n_kj[i] <- a_n_kj + ### intercept

### Climate

b.map_n_kj * map[i] +

## Geology effect

b.basalt_n_kj * basalt[i]

# residuals (for posterior predictive check)

res.n_kj[i] <- pow((n_kj[i] - mu_n_kj[i]),2)

n_kj.new[i] ~ dnorm(mu_n_kj[i], prec_n_kj)

res.n_kj.new[i] <- pow((n_kj.new[i] - mu_n_kj[i]),2)

# log-lokelihood (for model comparison)

n_loglik[i] <- logdensity.norm(n_kj[i], mu_n_kj[i], prec_n_kj)

# Submodel Soil P

p_kj[i] ~ dnorm(mu_p_kj[i], prec_p_kj)

mu_p_kj[i] <- a_p_kj + ### intercept

### Climate

b.map_p_kj * map[i] +

## Geology effect

b.basalt_p_kj * basalt[i]

# residuals (for posterior predictive check)

res.p_kj[i] <- pow((p_kj[i] - mu_p_kj[i]),2)

p_kj.new[i] ~ dnorm(mu_p_kj[i], prec_p_kj)

res.p_kj.new[i] <- pow((p_kj.new[i] - mu_p_kj[i]),2)

# log-lokelihood (for model comparison)

p_loglik[i] <- logdensity.norm(p_kj[i], mu_p_kj[i], prec_p_kj)

# Submodel Soil C:N

cn[i] ~ dnorm(mu_cn[i], prec_cn)

mu_cn[i] <- a_cn + ### intercept

### Climate

b.map_cn * map[i] +

b.mat_cn * mat[i] +

## Geology effect

b.basalt_cn * basalt[i]

# residuals (for posterior predictive check)

res.cn[i] <- pow((cn[i] - mu_cn[i]),2)

cn.new[i] ~ dnorm(mu_cn[i], prec_cn)

res.cn.new[i] <- pow((cn.new[i] - mu_cn[i]),2)

# log-lokelihood (for model comparison)

cn_loglik[i] <- logdensity.norm(cn[i], mu_cn[i], prec_cn)

# Submodel Soil K

k[i] ~ dnorm(mu_k[i], prec_k)

mu_k[i] <- a_k + ### intercept

### Climate

b.map_k * map[i] +

b.mat_k * mat[i] +

## Geology effect

b.basalt_k * basalt[i]

# residuals (for posterior predictive check)

res.k[i] <- pow((k[i] - mu_k[i]),2)

k.new[i] ~ dnorm(mu_k[i], prec_k)

res.k.new[i] <- pow((k.new[i] - mu_k[i]),2)

# log-lokelihood (for model comparison)

k_loglik[i] <- logdensity.norm(k[i], mu_k[i], prec_k)

# Submodel Soil Na

na[i] ~ dnorm(mu_na[i], prec_na)

mu_na[i] <- a_na + ### intercept

### Climate

b.mat_na * mat[i] +

## Geology effect

b.basalt_na * basalt[i]

# residuals (for posterior predictive check)

res.na[i] <- pow((na[i] - mu_na[i]),2)

na.new[i] ~ dnorm(mu_na[i], prec_na)

res.na.new[i] <- pow((na.new[i] - mu_na[i]),2)

# log-lokelihood (for model comparison)

na_loglik[i] <- logdensity.norm(na[i], mu_na[i], prec_na)

}

########

# Priors #

########

phi ~ dgamma(.1,.1)

# Species-specific Intercepts

for(sp in 1:nspp_intercept){

a_hr[sp] ~ dnorm(0,.001)

a_availN[sp] ~ dnorm(0,.001)

a_total_N[sp] ~ dnorm(0,.001)

}

# Soil intercepts

a_n_kj ~ dnorm(0,.001)

a_p_kj ~ dnorm(0,.001)

a_cn ~ dnorm(0,.001)

a_k ~ dnorm(0,.001)

a_na ~ dnorm(0,.001)

# Genus-specific Slopes

for(sp in 1:ngenus_slope){

# Slopes Herbivory model

### Climate

b.mat_hr[sp] ~ dnorm(0,1.0E-6)

### Soil

b.cn_hr[sp] ~ dnorm(0,1.0E-6)

### Foliage

b.totalN_hr[sp] ~ dnorm(0,1.0E-6)

###Geology

b.basalt_hr[sp] ~ dnorm(0,1.0E-6)

# Slopes Foliage model

# N digestibility

### Climate

b.mat_availN[sp] ~ dnorm(0,1.0E-6)

### Soil

b.p_kj_availN[sp] ~ dnorm(0,1.0E-6)

b.na_availN[sp] ~ dnorm(0,1.0E-6)

###Geology

b.basalt_availN[sp] ~ dnorm(0,1.0E-6)

# total_N

### Climate

b.map_total_N[sp] ~ dnorm(0,1.0E-6)

b.mat_total_N[sp] ~ dnorm(0,1.0E-6)

###Geology

b.basalt_total_N[sp] ~ dnorm(0,1.0E-6)

}

# Slopes soil

# Submodel soil N

b.map_n_kj ~ dnorm(0,1.0E-6)

b.basalt_n_kj ~ dnorm(0,1.0E-6)

# Submodel soil P

b.map_p_kj ~ dnorm(0,1.0E-6)

b.basalt_p_kj ~ dnorm(0,1.0E-6)

# Submodel soil C:N

b.map_cn ~ dnorm(0,1.0E-6)

b.mat_cn ~ dnorm(0,1.0E-6)

b.basalt_cn ~ dnorm(0,1.0E-6)

# Submodel soil K

b.map_k ~ dnorm(0,1.0E-6)

b.mat_k ~ dnorm(0,1.0E-6)

b.basalt_k ~ dnorm(0,1.0E-6)

# Submodel soil Na

b.mat_na ~ dnorm(0,1.0E-6)

b.basalt_na ~ dnorm(0,1.0E-6)

# Precision hierarchical models

prec_avail ~ dgamma(0.01, 0.01)

prec_total_N ~ dgamma(0.01, 0.01)

prec_n_kj ~ dgamma(0.01, 0.01)

prec_p_kj ~ dgamma(0.01, 0.01)

prec_cn ~ dgamma(0.01, 0.01)

prec_k ~ dgamma(0.01, 0.01)

prec_na ~ dgamma(0.01, 0.01)

################

# Random effects #

################

# herbivory model

for(s in 1:nsites){ r.site[s] ~ dnorm(0, tau.site) }

sd.site <- 1 / sqrt(tau.site)

tau.site ~ dgamma(0.01, 0.01)

# N digestibility

for(i in 1:nsites){ eps_availN[i] ~ dnorm(0, tau_availN) }

tau_availN ~ dgamma(0.01, 0.01)

sd_availN <- 1 / sqrt(tau_availN)

# total_N

for(i in 1:nsites){ eps_total_N[i] ~ dnorm(0, tau_total_N) }

tau_total_N ~ dgamma(0.01, 0.01)

sd_total_N <- 1 / sqrt(tau_total_N)

###########################################

# Sum of residuals (for Posterior Predictive Checks) #

###########################################

fit.herb <- sum(res.herb[])

fit.herb.new <- sum(res.herb.new[])

fit.availN <- sum(res.availN[])

fit.availN.new <- sum(res.availN.new[])

fit.total_N <- sum(res.total_N[])

fit.total_N.new <- sum(res.total_N.new[])

fit.n_kj <- sum(res.n_kj[])

fit.n_kj.new <- sum(res.n_kj.new[])

fit.p_kj <- sum(res.p_kj[])

fit.p_kj.new <- sum(res.p_kj.new[])

fit.cn <- sum(res.cn[])

fit.cn.new <- sum(res.cn.new[])

fit.k <- sum(res.k[])

fit.k.new <- sum(res.k.new[])

fit.na <- sum(res.na[])

fit.na.new <- sum(res.na.new[])
