# Supplementary material for: Relationships between abiotic factors, foliage chemistry and herbivory in a tropical montane ecosystem
Source: Oecologia. 2024 Oct 25;206(3-4):293–304. doi: 10.1007/s00442-024-05630-y (PMC11599541; doi:10.1007/s00442-024-05630-y)
Supplement: Supplementary file 2 — Supplementary file2 (DOCX 6286 KB) [file 442_2024_5630_MOESM2_ESM.docx]

# Supplementary Materials

### Variable selection and model structure

We performed a pairwise correlation analysis to evaluate the degree of association among the potential predictors examined in this study (Figure S2). Notably, we found a high correlation between dry matter and N digestibility (*r* = 0.98) and between basalt and granite parent materials (*r* = -0.77). Since the N digestibility index already encompassed information about changes in dry matter digestibility, we proceeded with only N digestibility for constructing the hierarchical model. Similarly, because 88% of the sites were either derived from basalt or granite, basaltic geological origin already included information about granite soils (i.e., basalt = 0). Therefore, we proceeded with basalt as a predictor in subsequent models. Further, we found a high correlation between basalt and soil P (*r* = 0.8). Since both parameters inform different aspects of resource availability gradients, we initially included both predictors in the foliage and herbivory models. However, we only retained the predictor that increased predictive accuracy following the variable selection approach described below.

Our model encompassed eight interconnected sub-models: five soil models (i.e., one for each element), two foliage models (i.e., total N and N digestibility), and one herbivory model. The propagation of uncertainty followed the directionality of the relationships among the components of the system, expressed by the hierarchical structure of the model (Figure 2). To achieve a more parsimonious model, we employed variable selection, aiming to maximise predictive accuracy while adhering to the principles of marginality (McCullagh, 2019). Variable selection was performed following the leave-one-out selection method using the “loo” package (Vehtari et al., 2020), an appropriate approach for variable selection in hierarchical models (Hooten and Hobbs, 2015).

Following the hierarchical interconnections within our model, we initiated the variable selection process by evaluating the impact of climate and geology on soil chemistry, followed by the effect of climate, geology, and soil nutrients on foliage chemistry, and then the effect of all the above factors on herbivory pressure. This sequential approach facilitated a coherent understanding of the direct and indirect influences of all predictors across various model components while reducing the probability of mistakenly eliminating a predictor with an important role in shaping the system. A summary variable selection table is provided in Supplementary Materials (Table S1).


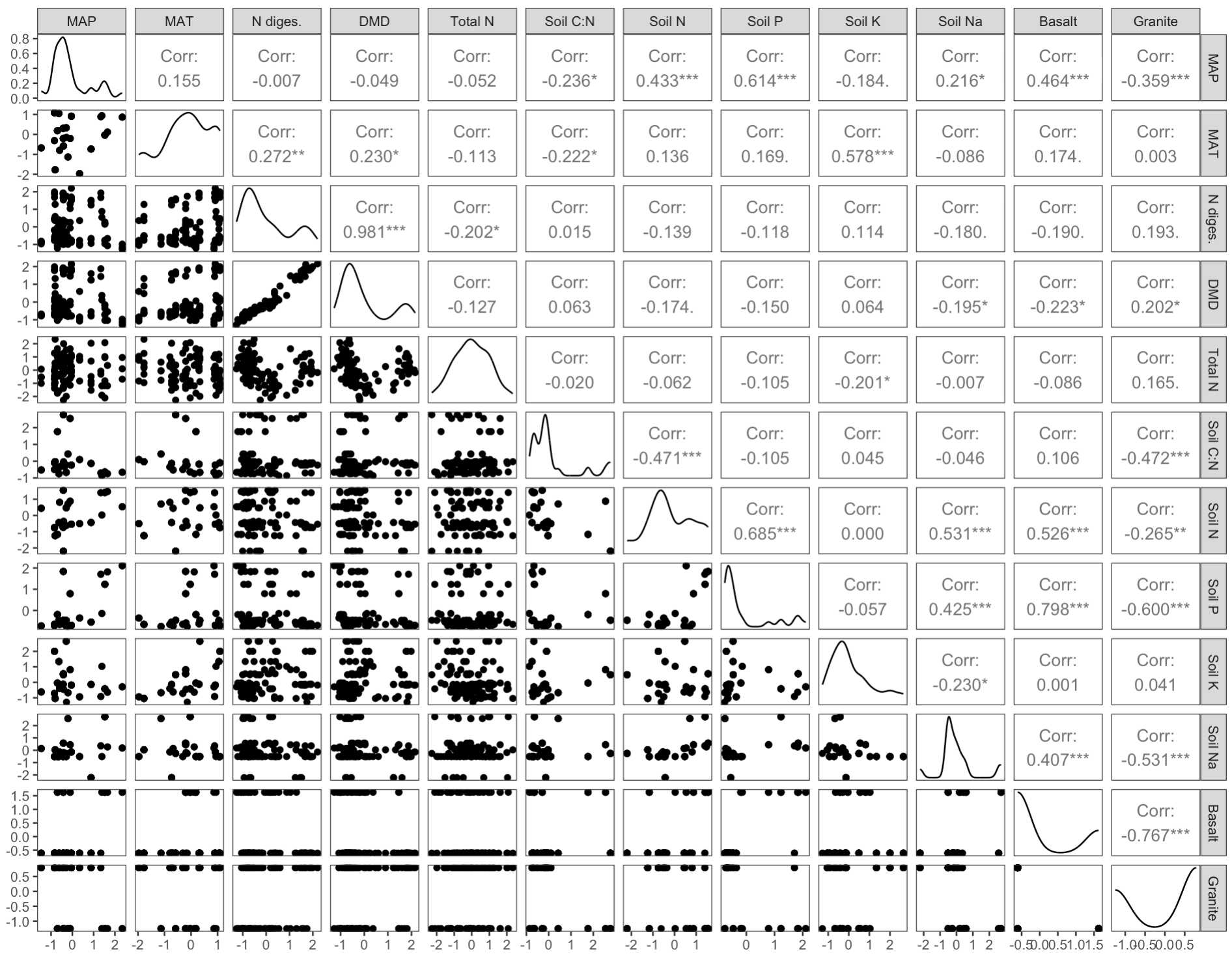


Figure S1. Pairwise correlation among potential model predictors. The main diagonal plots show the distribution of values for each variable. Scatterplots below the diagonal represent the raw relationship between pairs of variables, while values above the diagonal indicate the level of correlation of such predictors, with the level of significance following traditional notation (*95%; **99%; ***99.99%).


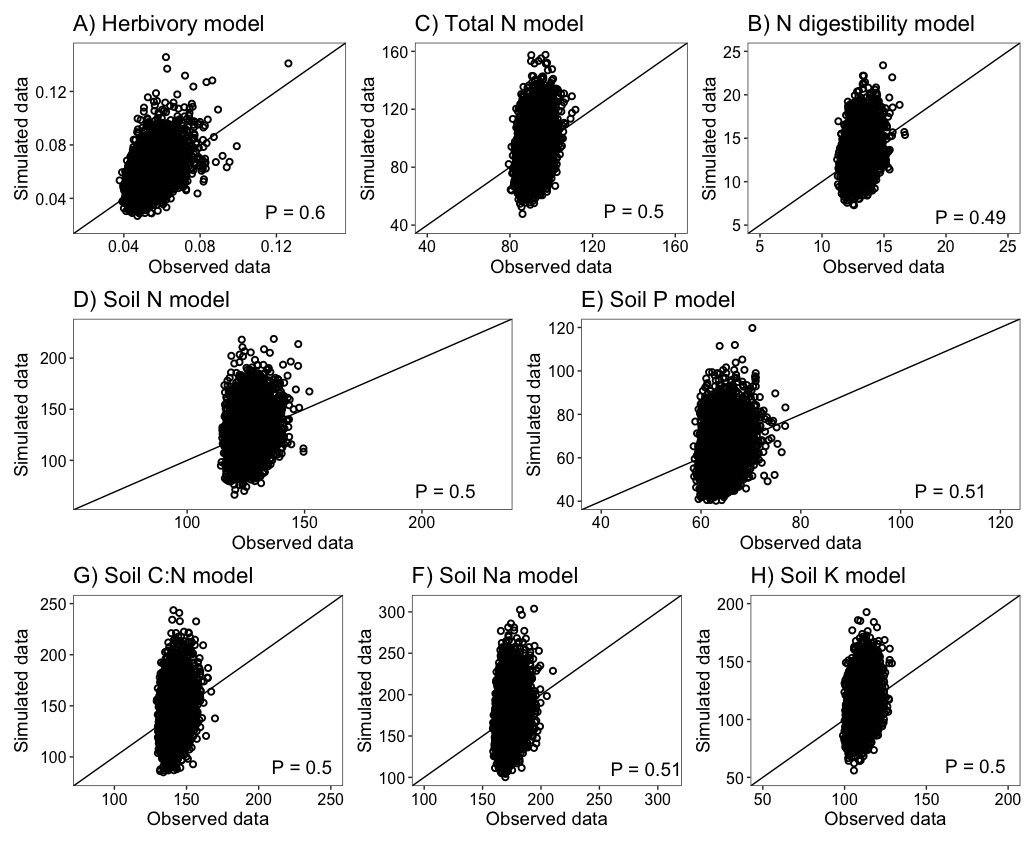


Figure S2. Posterior predictive check and Bayesian p-value for the final sub-models.


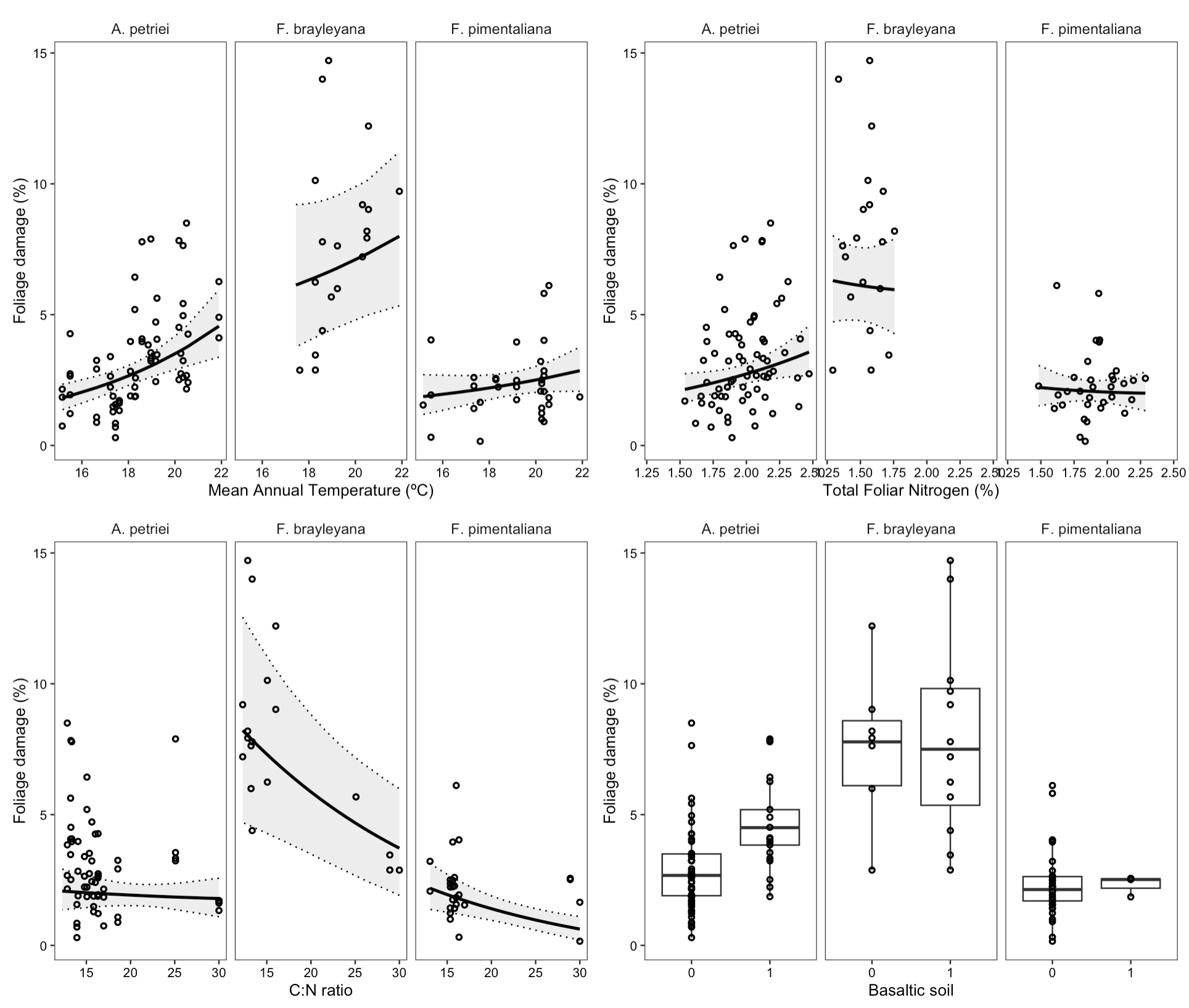


Figure S3. Patterns in herbivory across selected gradients. Predictive line shows the average effect with the shade area representing the 89% CI.


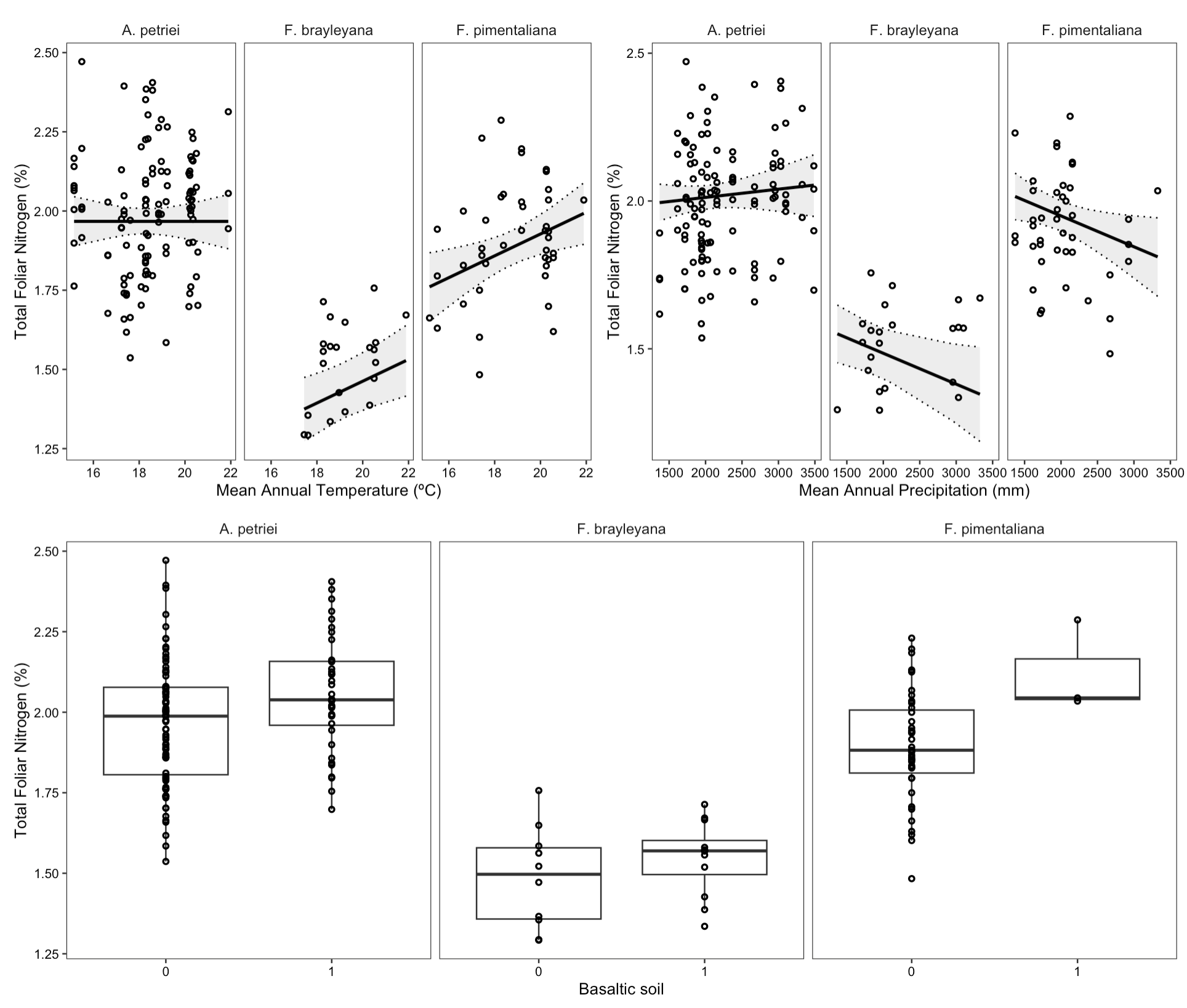


Figure S4. Patterns in foliar total N across selected gradients. Predictive line shows the average effect with the shade area representing the 89% CI.


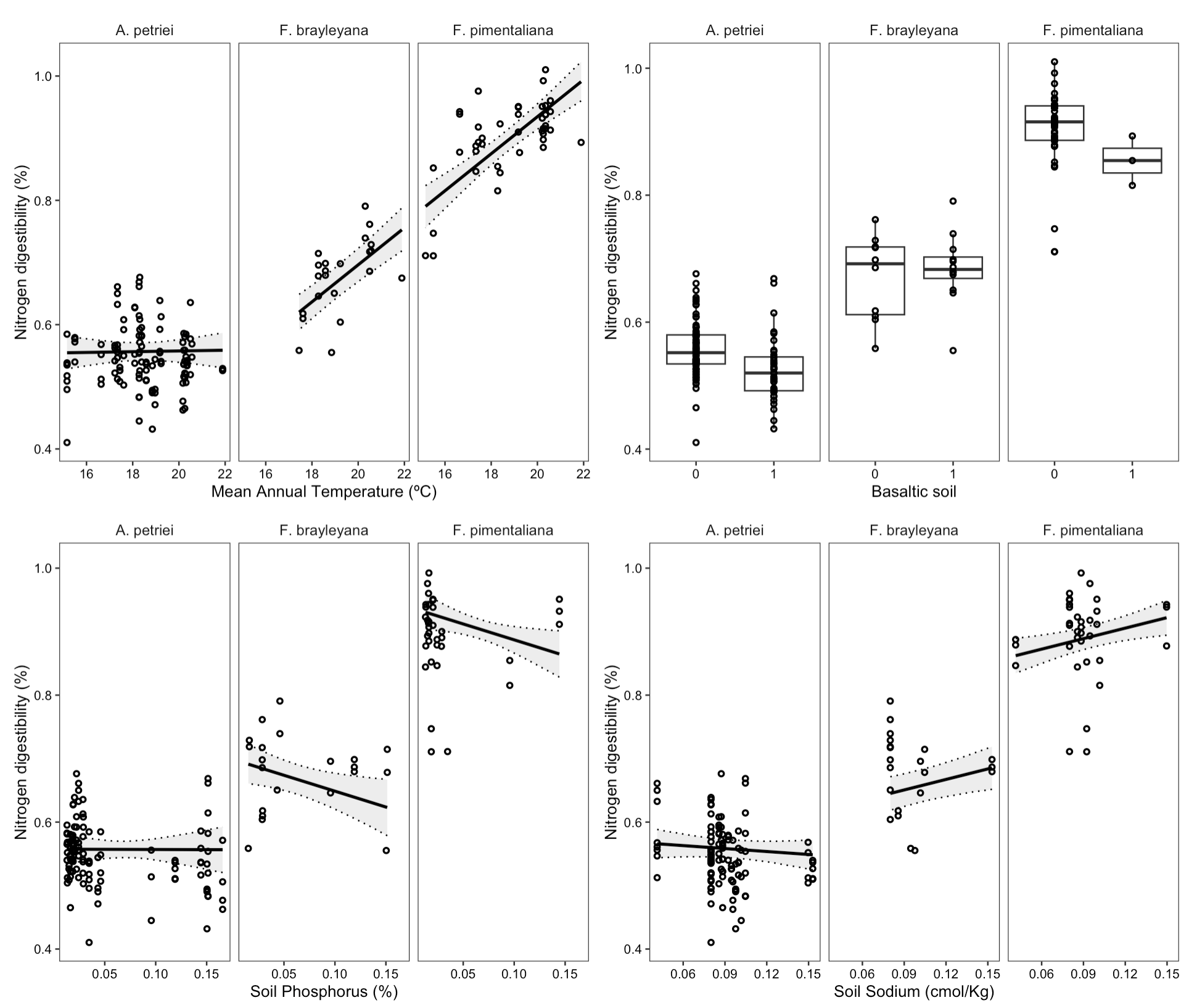


Figure S5. Patterns in foliar N digestibility across selected gradients. Predictive line shows the average effect with the shade area representing the 89% CI.


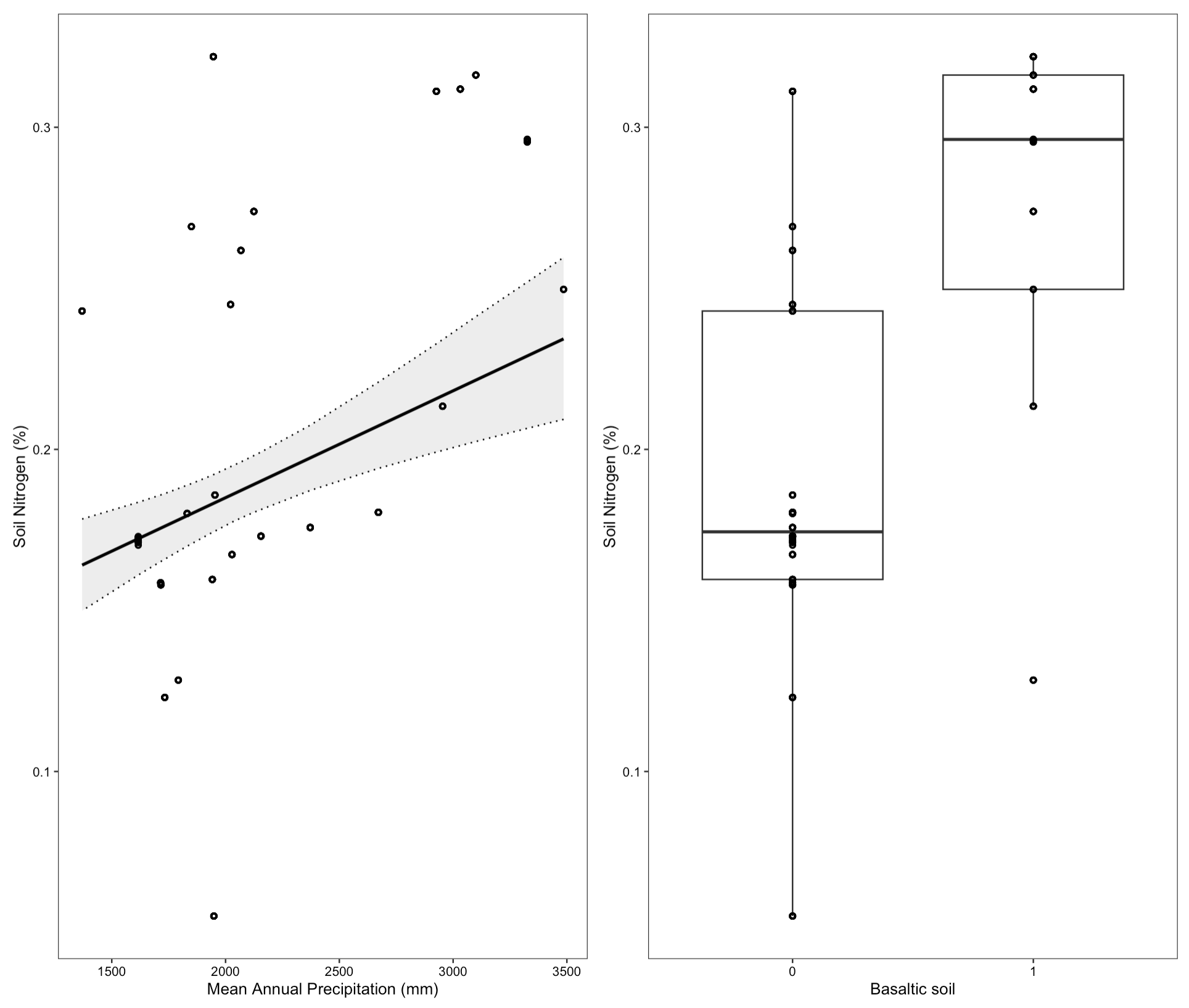


Figure S6. Patterns in soil N across selected gradients. Predictive line shows the average effect with the shade area representing the 89% CI.


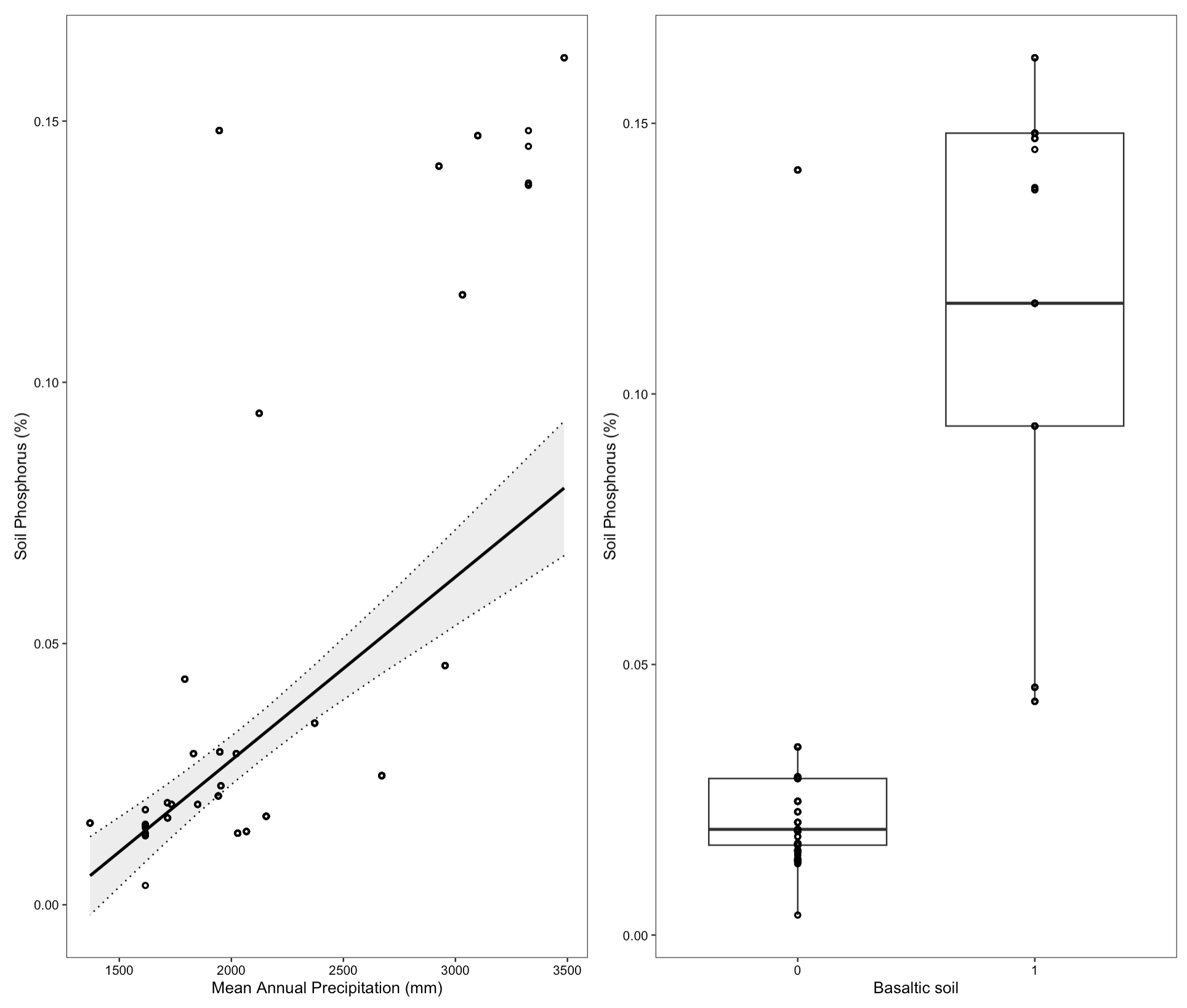


Figure S7. Patterns in soil P across selected gradients. Predictive line shows the average effect with the shade area representing the 89% CI.


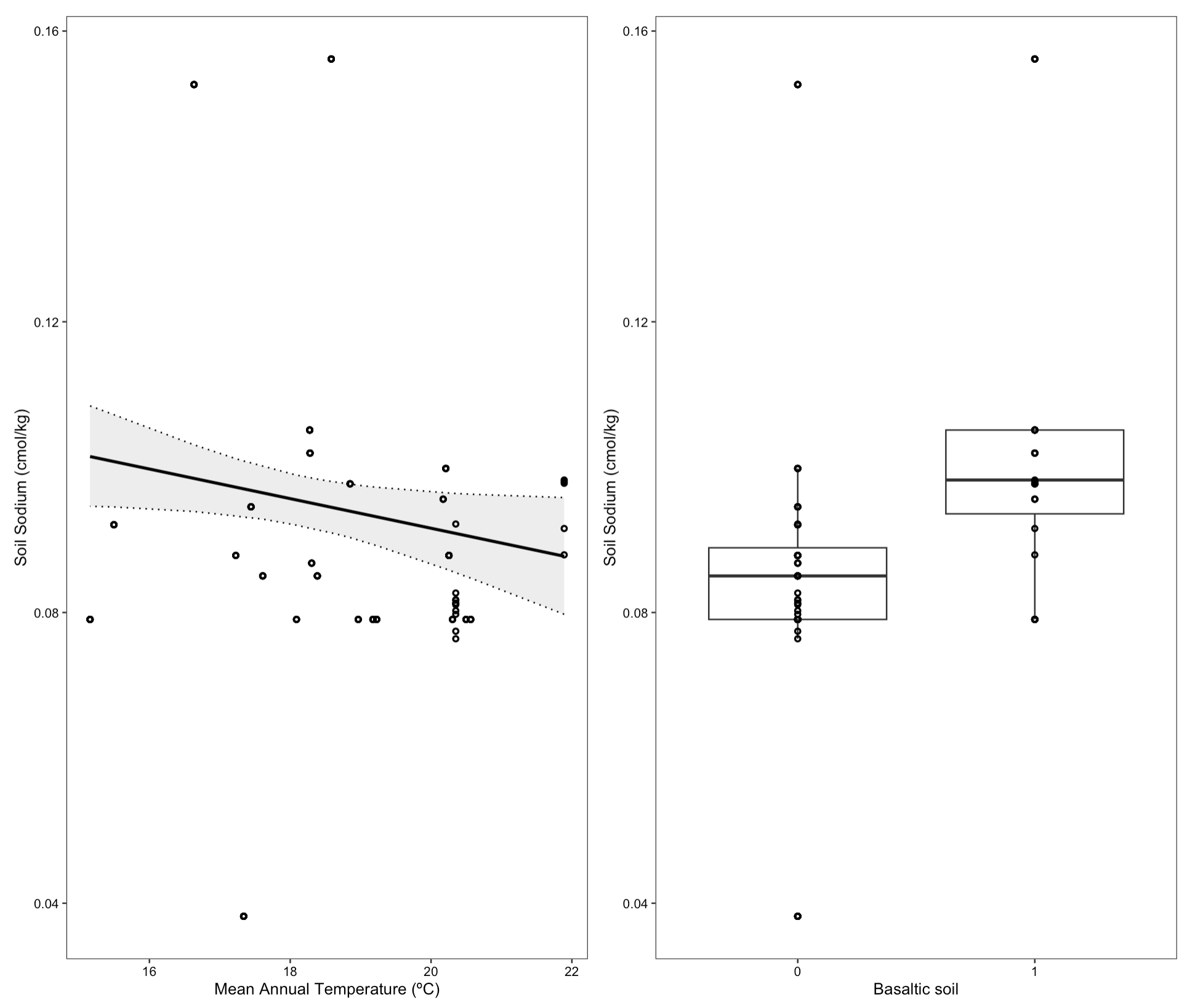


Figure S8. Patterns in soil Na across selected gradients. Predictive line shows the average effect with the shade area representing the 89% CI.


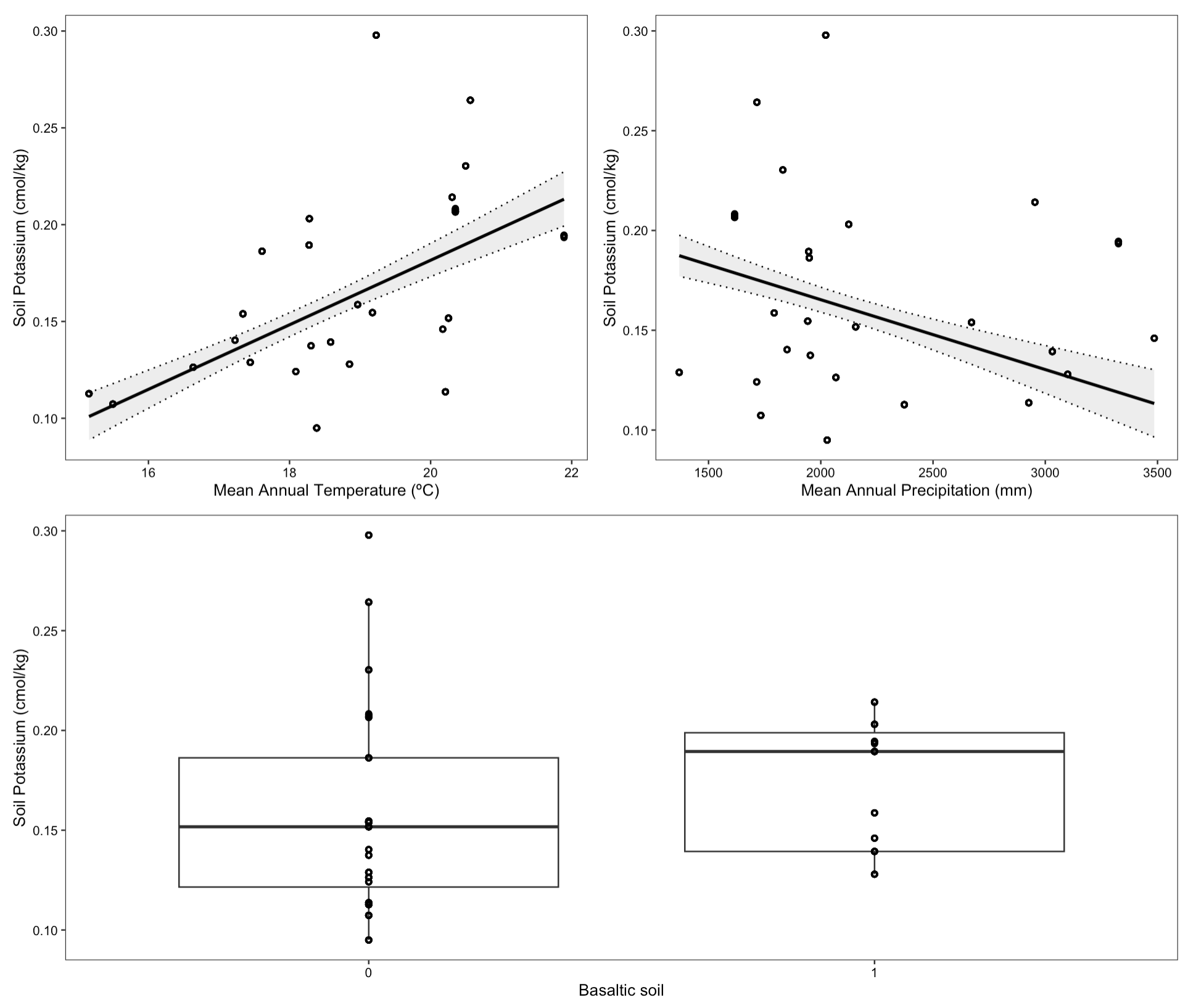


Figure S9. Patterns in soil K across selected gradients. Predictive line shows the average effect with the shade area representing the 89% CI.


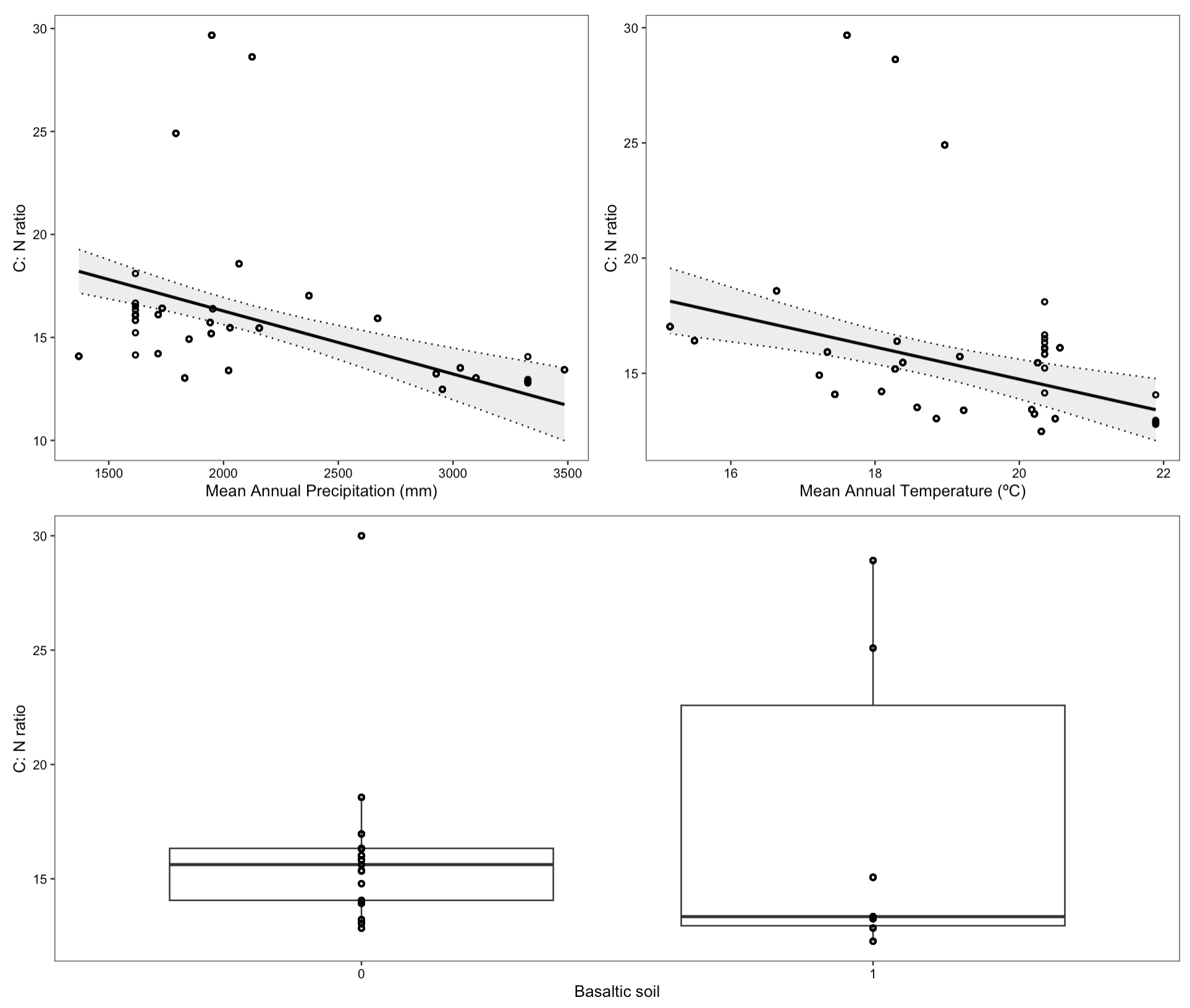


Figure S10. Patterns in soil C:N ratio across selected gradients. Predictive line shows the average effect with the shade area representing the 89% CI.

Table S1. Variable selection results comparing the expected predictive accuracy (theoretical expected log pointwise predictive density or ELPD) between most complex version of each sub-model and the stepwise removal of predictors with marginal effect. A positive difference in predictive value (ΔELPD) suggest that the removal of that predictor increased the predictive accuracy of the model. For sub-models with only “Most Complex” competing model, no variable removal improved predictive accuracy, meaning that the most complex model performed best.

| Hierarchy | Sub-model | Competing model | ELPD LOO | ΔELPD |
| --- | --- | --- | --- | --- |
| Soil | Soil Na | Most Complex | -263.3 |  |
|  |  | Remove MAP | -262.3 | 1 |
|  | Soil K | Most Complex | -222.6 |  |
|  | Soil CN | Most Complex | -244.3 |  |
|  | Soil P | Most Complex | -174.1 |  |
|  |  | Remove MAT | -173.9 | 0.2 |
|  | Soil N | Most Complex | -232.9 |  |
|  |  | Remove MAT | -232.3 | 0.6 |
| Foliage | Total N | Most Complex | -209.9 |  |
|  |  | Remove Soil Na | -208.1 | 1.8 |
|  |  | Remove Soil K | -207 | 1.1 |
|  |  | Remove Soil CN | -205.5 | 1.5 |
|  |  | Remove Soil N | -204.2 | 1.3 |
|  |  | Remove Soil P | -204 | 0.2 |
|  | N digestibility | Most Complex | -38 |  |
|  |  | Remove Soil K | -36.7 | 1.3 |
|  |  | Remove Soil CN | -35.5 | 1.2 |
|  |  | Remove MAP | -33.9 | 1.6 |
|  |  | Remove Soil N | -33.8 | 0.1 |
| Herbivory | Damage | Most Complex | 435.7 |  |
|  |  | Remove Soil P | 437 | 1.3 |
|  |  | Remove N digestibility | 441.5 | 4.5 |
|  |  | Remove Soil Na | 442.7 | 1.2 |
|  |  | Remove Soil K | 442.8 | 0.1 |
|  |  | Remove MAP | 443.6 | 0.8 |
|  |  | Remove Soil N | 444 | 0.4 |

Table S2. Model intercepts. Values are reported as the mean and 89% credible interval (CI).

| **Model** | **Species** | **Mean** | **Lower CI** | **Upper CI** |
| --- | --- | --- | --- | --- |
| Herbivory (%) | *Alphitonia petriei* | 2.9 | 2.5 | 3.3 |
| Herbivory (%) | *Flindersia brayleyana* | 6.6 | 4.3 | 9.4 |
| Herbivory (%) | *Flindersia pimentaliana* | 2.3 | 1.9 | 2.7 |
| N digestibility (Fraction 0-1) | *Alphitonia petriei* | 0.56 | 0.54 | 0.57 |
| N digestibility (Fraction 0-1) | *Flindersia brayleyana* | 0.66 | 0.63 | 0.68 |
| N digestibility (Fraction 0-1) | *Flindersia pimentaliana* | 0.89 | 0.88 | 0.91 |
| Total N (%) | *Alphitonia petriei* | 1.97 | 1.93 | 2.01 |
| Total N (%) | *Flindersia brayleyana* | 1.42 | 1.33 | 1.51 |
| Total N (%) | *Flindersia pimentaliana* | 1.88 | 1.83 | 1.93 |
| Soil N (%) |  | 0.19 | 0.18 | 0.2 |
| Soil P (%) |  | 0.034 | 0.03 | 0.039 |
| Soil C: N (ratio) |  | 15.73 | 15.07 | 16.39 |
| Soil K (cmol/kg) |  | 0.16 | 0.153 | 0.165 |
| Soil Na (cmol/kg) |  | 0.084 | 0.081 | 0.088 |

Table S3. Summary of sample size and treatment of the datasets used in each sub-model.

| Sub-model | Scale | Data collected | Data treatment | Sample size for the model |
| --- | --- | --- | --- | --- |
| Soil | Site | Three soil samples were collected at three different depths across 22 sites. The three missing sites were stochastically imputed based on the overarching biogeochemical dynamics predicted by the model | Chemical concentration for each soil element was averaged across the vertical gradient | 22 |
| Foliage | Tree | 179 canopy trees were collected for three different species | Chemical assays were performed on a representative subsample (33%) selected based on near-infrared spectroscopy techniques. Chemical profiles for the remaining samples were predicted using calibrated equations (see Materials and methods) | 179 |
| Herbivory | Tree | An average of 20 individual leaves were sampled across 120 canopy trees | Herbivory damage was averaged at the tree level based on the 20 individual measures per tree | 120 |
| Climate | Site | Data retrieved from spatial climate layers | Mean annual temperature and precipitation were extracted from spatial layers adjusted for the region (see Materials and methods) | 25 |

Table S4. Site description.

| Site ID | Geology | Elevation (m) | Latitude | Longitude | Mean Annual Precipitation (mm) | Mean Annual Temperature (˚C) |
| --- | --- | --- | --- | --- | --- | --- |
| AU10A | basalt | 940 | -17.7062 | 145.5267 | 1946.19382 | 18.2770004 |
| AU4A | basalt | 410 | -17.608226 | 145.769781 | 3325.91078 | 21.8910008 |
| AU6A | basalt | 630 | -17.673722 | 145.71513 | 2953.89001 | 20.3050003 |
| AU7A | granite | 690 | -17.631963 | 145.693306 | 2926.42775 | 20.2099991 |
| AU7B | basalt | 750 | -17.618911 | 145.68341 | 3485.48296 | 20.1720009 |
| AU8A | basalt | 850 | -17.603419 | 145.632515 | 3099.89512 | 18.8500004 |
| AU8B | basalt | 820 | -17.597161 | 145.646271 | 3031.61144 | 18.5830002 |
| AUAC | basalt | 830 | -17.743289 | 145.534109 | 2124.41302 | 18.2810001 |
| AUAD | basalt | 760 | -17.770123 | 145.547467 | 1792.43365 | 18.9659996 |
| AUSR | rhyolite | 900 | -17.7154 | 145.5267 | 2028.3956 | 18.3850002 |
| CU10A | granite | 1050 | -16.556704 | 145.276664 | 2671.55869 | 17.3390007 |
| CU11 | granite | 1120 | -16.529514 | 145.284013 | 1732.86775 | 15.4960003 |
| CU12A | granite | 1190 | -16.516485 | 145.275519 | 2372.22501 | 15.1569996 |
| CU6A | granite | 630 | -16.577398 | 145.309423 | 1616.30773 | 20.3509998 |
| CU8A | granite | 805 | -16.584539 | 145.303007 | 2155.69822 | 20.2539997 |
| G5 | rhyolite | 1040 | -17.688477 | 145.507957 | 1948.67603 | 17.6100006 |
| HRG1 | rhyolite | 1160 | -17.454248 | 145.476429 | 2067.48736 | 16.6340008 |
| HRG3 | granite | 1230 | -17.286008 | 145.426695 | 1369.47718 | 17.4430008 |
| HRG3A | granite | 1170 | -17.316392 | 145.419989 | 1849.89389 | 17.2269993 |
| LU10 | granite | 990 | -17.102851 | 145.590055 | 1941.90305 | 19.1749992 |
| LU11 | granite | 1090 | -17.092501 | 145.618886 | 1953.35974 | 18.3050003 |
| LU12 | granite | 1180 | -17.096672 | 145.577881 | 1714.58576 | 18.0890007 |
| LU7 | granite | 705 | -17.124248 | 145.629435 | 1831.17529 | 20.4969997 |
| LU8 | granite | 760 | -17.117477 | 145.602962 | 1715.59607 | 20.5629997 |
| LU9 | granite | 900 | -17.103734 | 145.611918 | 2022.01865 | 19.2280006 |

Table S5. Trees sampled per plot.

| Site ID | Alphitonia petriei | Flindersia brayleyana | | Flindersia pimentaliana | |
| --- | --- | --- | --- | --- | --- |
| AU10A | 8 | 2 | 0 | |  |
| AU4A | 3 | 1 | 1 | |  |
| AU6A | 4 | 2 | 0 | |  |
| AU7A | 5 | 0 | 3 | |  |
| AU7B | 4 | 0 | 0 | |  |
| AU8A | 5 | 1 | 0 | |  |
| AU8B | 5 | 3 | 0 | |  |
| AUAC | 3 | 2 | 2 | |  |
| AUAD | 4 | 1 | 0 | |  |
| AUSR | 5 | 0 | 2 | |  |
| CU10A | 8 | 0 | 3 | |  |
| CU11 | 5 | 0 | 3 | |  |
| CU12A | 8 | 0 | 1 | |  |
| CU6A | 5 | 0 | 6 | |  |
| CU8A | 6 | 0 | 5 | |  |
| G5 | 4 | 2 | 2 | |  |
| HRG1 | 4 | 0 | 3 | |  |
| HRG3 | 4 | 1 | 3 | |  |
| HRG3A | 4 | 0 | 0 | |  |
| LU10 | 4 | 0 | 4 | |  |
| LU11 | 5 | 0 | 0 | |  |
| LU12 | 4 | 0 | 0 | |  |
| LU7 | 3 | 3 | 0 | |  |
| LU8 | 2 | 2 | 3 | |  |
| LU9 | 3 | 2 | 1 | |  |
